# Synthesis and Anticancer Activity of Glucosylated Podophyllotoxin Derivatives Linked via 4*β*-Triazole Rings

**DOI:** 10.3390/molecules181113992

**Published:** 2013-11-13

**Authors:** Cheng-Ting Zi, Feng-Qing Xu, Gen-Tao Li, Yan Li, Zhong-Tao Ding, Jun Zhou, Zi-Hua Jiang, Jiang-Miao Hu

**Affiliations:** 1 State Key Laboratory of Phytochemistry and Plant Resources in West China, Kunming Institute of Botany, Chinese Academy of Sciences, Kunming 650201, China; E-Mails: zict@sina.cn (C.-T.Z.); hfglnds@sina.com (F.-Q.X.); ligentao@mail.kib.ac.cn (G.-T.L.); liyanb@mail.kib.ac.cn (Y.L.); jzhou@mail.kib.ac.cn (J.Z.); 2 Key Laboratory of Medicinal Chemistry for Natural Resource, Yunnan University, Kunming 650091, China; E-Mail: ztding@ynu.edu.cn; 3 Department of Chemistry, Lakehead University, 955 Oliver Road, Thunder Bay, ON P7B 5E1, Canada

**Keywords:** podophyllotoxin, 4*β*-triazole ring, click chemistry, anticancer activity

## Abstract

A series of 4*β*-triazole-linked glucose podophyllotoxin conjugates have been designed and synthesized by employing a click chemistry approach. All the compounds were evaluated for their anticancer activity against a panel of five human cancer cell lines (HL-60, SMMC-7721, A-549, MCF-7, SW480) using MTT assays. Most of these triazole derivatives have good anticancer activity. Among them, compound **35** showed the highest potency against all five cancer cell lines tested, with IC_50_ values ranging from 0.59 to 2.90 μM, which is significantly more active than the drug etoposide currently in clinical use. Structure-activity relationship analysis reveals that the acyl substitution on the glucose residue, the length of oligoethylene glycol linker, and the 4'-demethylation of podophyllotoxin scaffold can significantly affect the potency of the anticancer activity. Most notably, derivatives with a perbutyrylated glucose residue show much higher activity than their counterparts with either a free glucose or a peracetylated glucose residue.

## 1. Introduction

**Figure 1:**
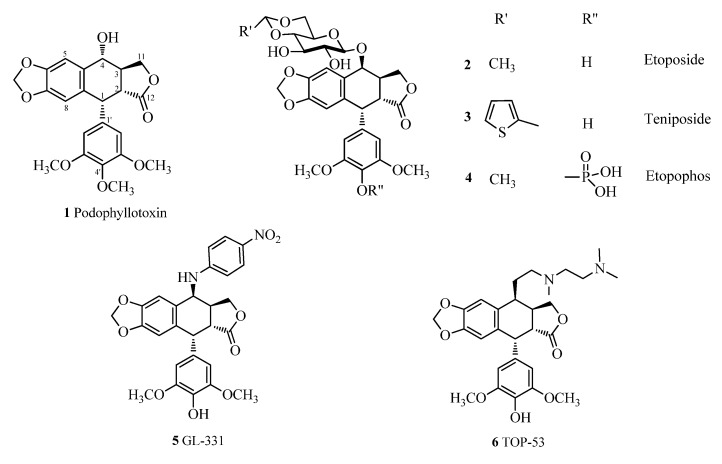
Structures of podophyllotoxin (**1**) and its semisynthetic derivatives.

Podophyllotoxin (**1**, Figure 1), which is a lignan mainly isolated from *Podophyllum peltatum* and *Podophyllum hexandrum* [1,2], shows strong cytotoxic activity against various cancer cell lines by inhibiting tubulin polymerization and preventing microtubule formation. Due to its complicated side effects such as nausea, vomiting, and damage of normal tissues, attempts to use podophyllotoxin in the treatment of human neoplasia have been mostly unsuccessful [3]. The unique cyclolignan scaffold of **1** has however drawn a lot of attention for the discovery and development of new anticancer agents. Extensive structural modifications, particularly at the C-4 and C-4' position of podophyllotoxin have led to the development of many semisynthetic derivatives of podophyllotoxin [4,5,6]. Among them, five semisynthetic derivatives, etoposide (**2**), teniposide (**3**), etopophos (**4**), GL-331 (**5**) and TOP-53 (**6**) (Figure 1) are currently used in the chemotherapy for a variety of cancers, including small-cell lung cancer, non-Hodgkin’s lymphoma, leukemia, Kaposi’s sarcoma, neurobslastoma and soft tissue sarcoma. These derivatives display binding activity to DNA topoisomerase II during the late S and early G2 cell cycle stages and are potent inhibitors of the enzyme [7,8,9,10,11,12,13,14]. Their anticancer activity proceeds through a mechanism of action entirely different from that of their parent compound podophyllotoxin (**1**). Etoposide (**2**), teniposide (**3**), and etopophos (**4**) are three semisynthetic glucosidic cyclic acetals of **1**, and in particular, etoposide (**2**) is considered to be one of the most successful pharmaceuticals derived from plants. Both GL-331 (**5**) and TOP-53 (**6**) are more active than etoposide (**2**) and are currently under clinical investigation [12].

Recently, novel podophyllotoxin hybrids obtained by covalently linking another biologically active molecule to podophyllotoxin have been reported. For example, thiocolchicine-podophyllotoxin conjugates were reported to have improved solubility and anticancer activity [15]. In addition, a series of conjugates of podophyllotoxin with 5-fluorouracil (5-FU) were reported to have better cytotoxic activity than VP-16 [16]. Structure-activity relationship (SAR) studies [17] have demonstrated that C-4 is the molecular area tolerable to significant structural diversification.

Chemotherapeutic agents such as the podophyllotoxin derivatives **2**–**4** are often associated with undesirable side effects and the development of multi-drug resistance by cancer cells. Thus, structural modification of podophyllotoxin for developing new antitumor drugs with increased selectivity and reduced toxicity is highly desirable. In recent years, the altered glucose metabolism in cancer cells has been explored for targeted cancer therapy [18]. Glucose is the main source of metabolic energy of animal cells, generating ATP through glycolysis and oxidative phosphorylation. Cancer cells are well known to display an enhanced uptake and consumption of glucose, which is metabolized primarily through the fermentative pathway instead of tricarboxylic acid cycle and oxidative phosphorylation in the mitochondria of normal cells [19]. The transport of glucose across the plasma membrane into the cytosol is mediated by a family of glucose transporters (GLUTs) [20,21]. Due to their enhanced glucose consumption, cancer cells generally express higher levels of GLUTs than normal cells [22]. For example, glucose transporter class 1 (GLUT1) has been found to be overexpressed in a variety of both solid and hematological malignancies such as large B-cell lymphoma, colorectal carcinomas, hepatocellular carcinoma, head and neck cancer, gastrointestinal stromal tumor (GIST), prostate carcinoma, thyroid carcinoma, renal cell cancer, lung cancer, pancreatic cancer, sarcomas and laryngeal carcinomas [19]. Thus, in this study we planned to covalently link a glucose residue to podopyllotoxin so the resulting cytotoxic agents may be preferably taken up by cancer cells through the mediation of GLUTs.

Recently, the click reaction has been widely used to covalently link two molecular fragments in creating a wide variety of drug-like molecules [23,24]. Typically, a terminal alkyne and an azide undergo a copper-catalyzed [3+2]-cycloaddition to generate a substituted 4*β*-triazole ring [25,26]. Podophyllotoxin derivatives containing the featured 4*β*-triazole ring have also been reported as potential DNA topoisomerase-II inhibitors [27], including a few compounds bearing a sugar residue [28]. Through click reactions we have now synthesized a series of glucose-podophyllotoxin conjugates in order to systematically study the effect of: (a) the length of the linker; (b) the substituent on the glucose; (c) the configuration of the anomeric carbon of glucose residue; and (d) the substituent on the 4-position of the E-ring of the podophyllotoxin scaffold on the anticancer activity of such conjugates. Herein we report the synthesis, the preliminary anticancer activity and the structure activity relationship of these conjugates.

## 2. Results and Discussion

### 2.1. Chemical Synthesis

**Scheme 1:**
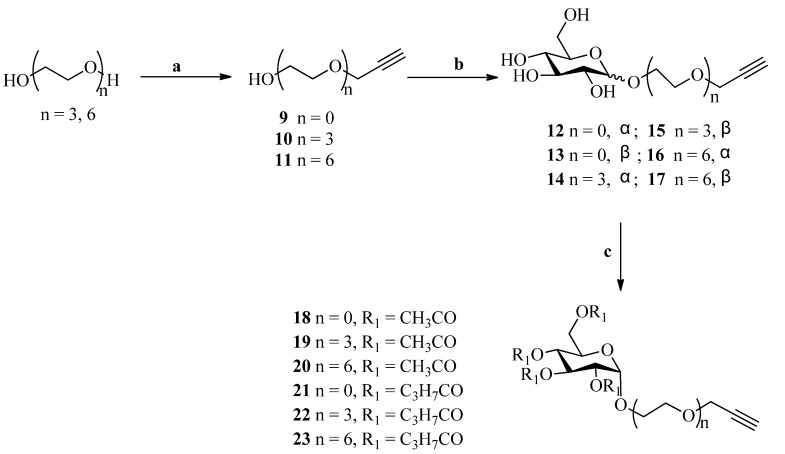
Synthesis of glucosylated terminal alkynes.

The preparation of terminal-alkynes is shown in Scheme 1. Compounds **10** and **11** were prepared in 70% yield by treatment of triethylene glycol and hexaethylene glycol with sodium hydride and propargyl bromide as previous described [29,30]. Fisher glycosylation of D-glucose with propargyl alcohols **9**–**11** in the presence of H_2_SO_4_-silica as a catalyst [31] provided the corresponding glycosides **12**–**17** as *α*/*β* mixtures. The *α*-isomer was usually obtained as the major isomer, and the *α*/*β* ratio was typically 6:1. The preparation of compounds **12** [32], **13** [32] and **15** [33] using a similar method has been reported in the literature. The major *α*-glycosides **12**, **14** and **16** were subjected to peracetylation and perbutyrylation with acetic anhydride and butyric anhydride in the presence of pyridine, to give the corresponding peracetylated (compounds **18**–**20**) and perbutyrylated products (compounds **21**–**23**), respectively, in 92%–98% yield. The preparation of compound **18** has been described in literature [34].

*Reagents and Reaction Conditions*: (**a**) propargyl bromide, sodium hydride, THF, reflux; (**b**) cat. H_2_SO_4_-silica, D-glucose; (**c**) acetic anhydride or *n*-butyric anhydride, pyridine, 0 °C–rt.

To introduce the azido functionality for the click-reaction, podophyllotoxin was readily converted to 4*β*-azido-4-deoxypodophyllotoxin (**7**) and 4*β*-azido-4-deoxy-4'-demethypodophyllotoxin (**8**) according to known procedures [35,36].

**Scheme 2:**
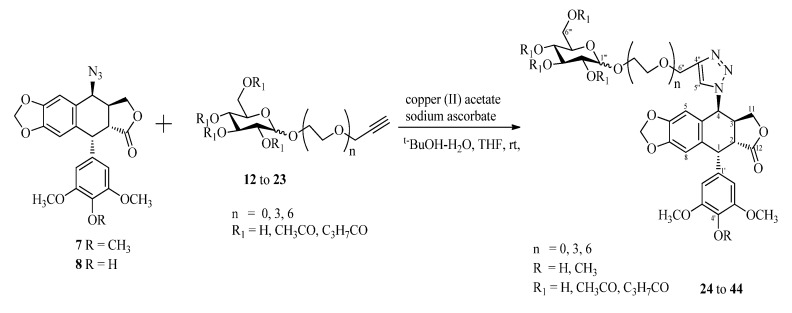
Click-chemistry strategy for the synthesis of 4*β*-triazole-linked glucose podophyllotoxin conjugates.

The glycosylated terminal alkynes **12**–**23** were allowed to react with azide **7** or **8** in the presence of copper (II) acetate and sodium ascorbate to yield a series of 4*β*-triazole-linked glucose-podophyllotoxin conjugates (Scheme 2, Table 1).

**Table 1:** A series of 4*β*-triazole-linked glucose-podophyllotoxin conjugates.

Compounds	n	R	R_1_	1'''–configuration	Yield% ^a^
**24**	0	CH_3_	H	*α*	98
**25**	0	CH_3_	CH_3_CO	*α*	92
**26**	0	CH_3_	C_3_H_7_CO	*α*	98
**27**	0	H	H	*α*	98
**28**	0	H	CH_3_CO	*α*	94
**29**	0	H	C_3_H_7_CO	*α*	96
**30**	3	CH_3_	H	*α*	91
**31**	3	CH_3_	CH_3_CO	*α*	94
**32**	3	CH_3_	C_3_H_7_CO	*α*	90
**33**	3	H	H	*α*	93
**34**	3	H	CH_3_CO	*α*	94
**35**	3	H	C_3_H_7_CO	*α*	92
**36**	6	CH_3_	H	*α*	91
**37**	6	CH_3_	CH_3_CO	*α*	91
**38**	6	CH_3_	C_3_H_7_CO	*α*	91
**39**	6	H	H	*α*	93
**40**	6	H	CH_3_CO	*α*	90
**41**	6	H	C_3_H_7_CO	*α*	97
**42**	0	H	H	*β*	97
**43**	3	H	H	*β*	92
**44**	6	H	H	*β*	94

^a^ Total isolated yield (%) was calculated after column chromatography.

All the products were characterized by ^1^H-NMR, ^13^C-NMR, ESI-MS, and HRESI-MS. In the ^1^H-NMR spectra, the proton at C-4 of 4*β-*triazole-substituted compounds appears as a doublet at *δ* 5.9–6.3 ppm, usually with a coupling constant *J*_3,4_ < 5.0 Hz, indicating a *cis*-relationship between H-3 and H-4. The formation of the triazole ring was confirmed by the resonance of its C^5"^-H signal (*δ* 7.8–8.2 ppm) in the aromatic region in the ^1^H-NMR spectra, which was further supported by two characteristic carbon signals at around 145 ppm and 126 ppm in the ^13^C-NMR spectra. The coupling constant of the anomeric proton of the glucose residue (*J*_1"',2"'_) is typically <4.0 Hz for the *α*-linkage and >7.6 Hz for the *β*-linkage. In addition, the anomeric carbon of an *α*-glucoside always has a lower chemical shift than the corresponding *β*-glucoside in the ^13^C-NMR spectra.

Poor water-solubility is a common problem in developing podophyllotoxin derivatives for therapeutic use. For the glucose-podophyllotoxin conjugates described in this study, compounds with a peracetylated or perbutyrylated glucose residue are slightly soluble in water. In general, the conjugates with a free glucose residue are soluble in water and methanol, while those with a peracetylated or a perbutyrylated glucose residue are more soluble in chloroform. For example, at room temperature compound **29** with a perbutyrylated glucose residue has a solubility of 1.7 mg/mL in water while compound **30** with a free glucose residue has a solubility of 13.3 mg/mL in water. Overall, the inclusion of the triazole-ring and the multiple triethylene glycol units improved the aqueous solubility of these compounds, and in the case when a free glucose residue is present, the compound becomes fairly soluble in water.

### 2.2. Evaluation of Biological Activity

All the 4*β*-triazole-linked glucose-podophyllotoxin conjugates **24**–**44** were tested for their anticancer activity against five human cancer cell lines, including HL-60 (leukemia), SMMC-7721 (hepatoma), A-549 (lung cancer), MCF-7 (breast cancer), and SW480 (colon cancer). Etoposide and cisplatin were taken as control drugs and the anticancer activity data are presented in Table 2. Our first observation is that compounds having a free glucose residue (compounds **24**, **27**, **30**, **33**, **36**, **39**, and **42**–**44**) mostly show weak activity (all having IC_50_ > 40 μM, except **30**), while several derivatives containing a peracetylated glucose residue (compounds **28**, **31** and **37**) show improved activity.

**Table 2:** *In vitro* anticancer activity (IC_50_, μM) of 4*β*-triazole-linked glucose-podophyllotoxin conjugates **24**–**44**.

Entry	IC_50_ (μM)				
	HL-60	SMMC-7721	A-549	MCF-7	SW480
**24**	>40	>40	>40	>40	>40
**25**	>40	>40	>40	>40	>40
**26**	>40	>40	>40	>40	>40
**27**	>40	>40	>40	>40	>40
**28**	6.77	27.17	>40	34.54	>40
**29**	0.80	3.03	4.05	3.90	4.36
**30**	13.13	19.12	20.17	22.33	28.58
**31**	12.12	38.33	33.73	29.41	>40
**32**	2.05	3.38	5.55	12.49	12.59
**33**	>40	>40	>40	>40	>40
**34**	>40	>40	>40	>40	>40
**35**	0.59	0.99	1.38	2.90	1.50
**36**	>40	>40	>40	>40	>40
**37**	12.82	26.31	31.84	38.69	40.00
**38**	1.66	3.79	4.55	5.20	6.54
**39**	>40	>40	>40	>40	>40
**40**	>40	>40	>40	>40	>40
**41**	15.07	17.65	26.68	28.77	28.05
**42**	>40	>40	>40	>40	>40
**43**	>40	>40	>40	>40	>40
**44**	>40	>40	>40	>40	>40
**Etoposide (2)**	0.31	8.12	11.92	32.82	17.11
**Cisplatin**	1.17	6.43	9.24	15.86	13.42

A group of compounds that display relatively high potency are those bearing the perbutyrylated glucose residue (compounds **29**, **32**, **35** and **38**). In most cases, derivatives with a perbutyrylated sugar residue are more active than those with a peracetylated glucose residue (**29** *vs*. **28**, **32** *vs*. **31**, **35** *vs*. **34**, **38** *vs*. **37**, and **40** *vs*. **41**), which in turn are more active than those with a free glucose residue (**28** *vs*. **27** and **37** *vs*. **36**).

The length of the linking spacer between the glucose moiety and the 1,2,3-triazole residue does not exhibit a uniform effect on the cytotoxic potency of these compounds. For example, among the set of compounds with a perbutyrylated sugar moiety and a methoxyl group at the C-4'-position (compounds **26**, **32** and **38**), compound **38** with the longest linking spacer (six ethylene glycol repeating units) is the most active compound against four out of five cancer cell lines tested, followed by **32** which has a shorter linker (three ethylene glycol repeating units), and **26** which has no linking spacer in-between is the least active. However, among the set of compounds with a perbutyrylated sugar moiety and a hydroxyl group at the C-4'-position (compounds **29**, **35** and **41**), compound **41** with the longest linking spacer is the least active while **35** with the shorter linker is the most active against all five cancer cell lines. Methylation of 4'-OH group in the E ring can lead to either increased or decreased activity, as indicated by the IC_50_ values of the pairs of compounds between **25/31/37** *vs*. **28/34/40** and **26/32/38** *vs*. **29/35/41**. The effect of glycosidic linkage on the cytotoxicity potency of these compounds can’t be concluded from this study because the exact IC_50_ values for the three pairs of *α/β* isomers (**27/33/39** *vs*. **42/43/44**) have not been determined (all having IC_50_ > 40 μM against all cancer cells tested).

Previously, Reddy and co-workers [28] reported the cytotoxic activity of several 4*β*-triazole-linked sugar-podophyllotoxin conjugates. The cytotoxic potency of compound **42** against A-549 cells reported in their paper was quite similar to our data, however, our findings that peracetylation and perbutyrylation of the glucose residue lead to increased activity are different from their observation [28]. Among all the synthesized compounds, several compounds (*i.e*., **32**, **35** and **38**) display significantly higher activity than etoposide (**2**), and in general derivatives containing a perbutyrylated D-glucose moiety are more active than other derivatives. *n*-Butyrate, a naturally occurring short-chain fatty acid, is a well known histone deacetylase (HDAC) inhibitor [37]. In recent years, HDAC inhibition has attracted much attention for the development of anticancer drugs because HDAC inhibitors are able to disrupt cell cycle progression or selectively induce apoptosis via depression of certain genes [38,39,40,41]. Thus, the butyrate species liberated from the hydrolysis of these butyrylated derivatives may have contributed to the apparently higher potency of these compounds. Further studies are needed in order to confirm the potential role of the butyryl substituents on the sugar residue.

## 3. Experimental

### 3.1. General

Melting points were uncorrected. MS data were obtained in the ESI mode on API Qstar Pulsar instrument. HRMS data were obtained in the ESI mode on LCMS-IT-TOF (Shimadzu, Kyoto, Japan). NMR spectra were acquired on Bruker AV-400 or DRX-500 or Bruker AVANCE Ш-600 (Bruker BioSpin GmbH, Rheinstetten, Germany) instruments, using tetramethylsilane (TMS) as an internal standard. Column chromatography (CC) was performed on flash silica gel (200–300 mesh; Qingdao Makall Group Co., Ltd., Qingdao, China). All reactions were monitored using thin-layer chromatography (TLC) on silica gel plates.

### 3.2. General Procedure for Fisher Glycosylation Catalyzed with H_2_SO_4_-on-Silica Gel (Preparation of **12**–**17**)

d-Glucose (5 mmol) was suspended in propargyl alcohol (25 mmol) and stirred at 65 °C. H_2_SO_4_-silica (25 mg) was added and stirring was continued until all solids had dissolved (~2.5 h). After cooling to room temperature, the reaction mixture was transferred to a short silica gel column and eluted using CHCl_3_/CH_3_OH = 9:1. The preparation of **12**, **13** and **15** using the same method has been reported in the literature [32,33].

*2-[2-[2-(2-Propyn-1-yloxy)ethoxy]ethoxy-α-d-glucopyranoside* (**14**). ^1^H-NMR (CD_3_OD, 400 MHz): *δ* 4.78 (d, 1H, *J* = 3.7 Hz, C^1^-H), 4.14 (d, 2H, *J* = 2.4 Hz, CH_2_-C≡C), 3.74 (d, 2H, *J* = 1.8 Hz), 3.61 (t, 6H, *J* = 1.1 Hz), 3.55 (t, 6H, *J* = 3.7 Hz), 3.33–3.34 (m, 4H), 3.25 (s, 4H), 2.82 (t, 1H, *J* = 2.3 Hz, C≡CH); ^13^C-NMR (CD_3_OD, 100 MHz): *δ* 100.2 (C-1), 80.6 (*C*≡CH), 76.1 (C≡*C*H), 75.1, 73.6, 71.7, 71.4, 71.3, 70.0, 68.1, 62.6 (C-6), 59.0 (*C*H_2_-C≡C); ESIMS: *m/z* 373 [M+Na]^+^.

*3,6,9,12,15,18-Hexaoxaheneicos-20-yn-1-yl-α-d-glucopyranoside* (**16**). ^1^H-NMR (CD_3_OD, 400 MHz): *δ* 4.82 (d, 1H, *J* = 3.6 Hz, C^1^-H), 4.18 (d, 2H, *J* = 2.0 Hz, CH_2_-C≡C), 3.78 (d, 1H, *J* = 11.4 Hz), 3.69 (t, 12H, *J* = 5.1 Hz), 3.64 (t, 12H, *J* = 2.0 Hz), 3.38–3.34 (m, 4H), 2.87 (s, 4H), 2.87 (t, 1H, *J* = 1.9 Hz, C≡CH); ^13^C-NMR (CD_3_OD, 100 MHz): *δ* 100.3 (C-1), 80.6 (*C*≡CH), 76.0 (C≡*C*H), 75.1, 73.6, 71.8, 71.5, 71.3, 70.1, 68.1, 62.6 (C-6), 59.0 (*C*H_2_-C≡C); ESIMS: *m/z* 505 [M+Na]^+^.

*3,6,9,12,15,18-Hexaoxaheneicos-20-yn-1-yl-β-d-glucopyranoside* (**17**). ^1^H-NMR (CD_3_OD, 400 MHz): *δ* 4.31 (d, 1H, *J* = 7.8 Hz, C^1^-H), 4.21 (d, 2H, *J* = 2.4 Hz, CH_2_-C≡C), 3.86 (d, 1H, *J* = 11.6 Hz), 3.68 (m, 12H), 3.66 (m, 12H), 3.21 (s, 4H), 2.88 (t, 1H, *J* = 2.4 Hz, C≡CH); ^13^C-NMR (CD_3_OD, 100 MHz): *δ* 104.4 (C-1), 80.6 (*C*≡CH), 77.9, 77.8, 75.9 (C≡*C*H), 71.6, 71.5, 71.5, 71.5, 71.3, 70.1, 70.0, 62.8, 59.0 (*C*H_2_-C≡C); ESIMS: *m/z* 505 [M+Na]^+^.

### 3.3. General Procedure for Acetylation and Butyrylation of the Glucoside (Preparation of **18**–**23**)

To a solution of a propargyl glycosides **12**–**17** (1 mmol) in pyridine (4.0 mL) at 0 °C, acetic anhydride (or butyryl anhydride) (4.0 mL) was added. The reaction mixture was stirred overnight until the starting material disappeared as indicated by TLC. The reaction mixture was diluted with water (20 mL) and extracted with ethyl acetate (3 × 20 mL). The organic layer was washed with 10% aqueous hydrochloric acid (20 mL) and brine (20 mL). The organic layer was dried over magnesium sulfate and evaporated to give a residue, which was chromatographed on silica gel with petroleum ether/acetone = 4:1→2:1 to give the peracetylated or perbutyrylated product. The preparation of **18** using the same method has been reported in the literature [34].

*2-[2-[2-(2-Propyn-1-yloxy)ethoxy]ethoxy-per-O-acetyl-α-d-glucopyranoside* (**19**). Yield: 96%. ^1^H-NMR (CD_3_OD, 400 MHz): *δ* 5.43 (t, 1H, *J* = 9.9 Hz, C^3^-H), 5.12 (d, 1H, *J* = 2.9 Hz, C^1^-H), 5.03 (t, 1H, *J* = 9.8 Hz, C^4^-H), 4.82 (d, 1H, *J* = 2.9 Hz, C^2^-H), 4.28–4.24 (m, 3H, C^6^-CHa, CH_2_-C≡C), 4.19–4.09 (m, 2H, C^6^-CHb, C^5^-H), 3.67 (m, 6H), 3.65 (m, 6H), 2.85 (s, 1H, C≡CH), 2.06 (s, 3H, COCH_3_), 2.05 (s, 3H, COCH_3_), 2.02 (s, 3H, COCH_3_), 1.99 (s, 3H, COCH_3_); ^13^C-NMR (CD_3_OD, 100 MHz): *δ* 172.3 (C=O), 171.6 (C=O), 171.6 (C=O), 171.3 (C=O), 97.0 (C-1), 80.7 (*C*≡CH), 76.0 (C≡*C*H), 72.1, 71.7, 71.6, 71.5, 71.4, 71.2, 70.1, 70.0, 68.7, 68.6, 63.2 63.2 (C-6), 59.0 (*C*H_2_-C≡C), 20.7 (CO*C*H_3_), 20.7 (CO*C*H_3_), 20.7 (CO*C*H_3_), 20.7 (CO*C*H_3_); ESIMS: *m/z* 541 [M+Na]^+^.

*3,6,9,12,15,18-Hexaoxaheneicos-20-yn-1-yl-per-O-butyryl-α-d-glucopyranoside* (**20**). Yield: 96%. ^1^H-NMR (CD_3_OD, 400 MHz): *δ* 5.43 (t, 1H, *J* = 9.8 Hz, C^3^-H), 5.12 (d, 1H, *J* = 3.5 Hz, C^1^-H), 5.03 (t, 1H, *J* = 9.7 Hz, C^4^-H), 4.82 (dd, 1H, *J* = 3.7 Hz, 10.3 Hz, C^2^-H), 4.27–4.24 (m, 3H, C^6^-CHa, CH_2_-C≡C), 4.17–4.12 (m, 2H, C^6^-CHb, C^5^-C), 3.65 (m, 12H), 3.63 (m, 12H), 2.86 (s, 1H, C≡CH), 2.06 (s, 3H, COCH_3_), 2.05 (s, 3H, COCH_3_), 2.02 (s, 3H, COCH_3_), 2.00 (s, 3H, COCH_3_); ^13^C-NMR (CD_3_OD, 100 MHz): *δ* 172.3 (C=O), 171.7 (C=O), 171.7 (C=O), 171.3 (C=O), 97.1 (C-1), 80.7 (*C*≡CH), 76.0 (C≡*C*H), 72.1, 71.7, 71.6, 72.6, 72.5, 71.4, 71.2, 70.1, 70.0, 63.2 (C-6), 59.0 (*C*H_2_-C≡C), 20.7 (CO*C*H_3_), 20.7 (CO*C*H_3_), 20.7 (CO*C*H_3_), 20.7 (CO*C*H_3_) ; ESIMS: *m/z* 673 [M+Na]^+^.

*2-Propyn-1-yl-per-O-butyryl-α-d-glucopyranoside* (**21**). Yield: 92%. ^1^H-NMR (CD_3_OD, 400 MHz): *δ* 5.45 (t, 1H, *J* = 9.9 Hz, C^3^-H), 5.29 (d, 1H, *J* = 3.6 Hz, C^1^-H), 5.12 (t, 1H, *J* = 9.8 Hz, C^4^-H), 4.90 (dd, 1H, *J* = 3.7 Hz, 10.3 Hz, C^2^-H), 4.35–4.24 (m, 3H, C^6^-CHa, CH_2_-C≡C), 4.13–4.09 (m, 2H, C^6^-CHb, C^5^-H), 2.91 (s, 1H, C≡CH), 2.34–2.24 (m, 2H, COCH_2_), 2.34-2.24 (m, 2H, COCH_2_), 2.34–2.24 (m, 2H, COCH_2_), 2.34–2.24 (m, 2H, COCH_2_), 1.66–1.56 (m, 2H, C*H*_2_CH_3_), 1.66–1.56 (m, 2H, C*H*_2_CH_3_), 1.66–1.56 (m, 2H, C*H*_2_CH_3_), 1.66–1.56 (m, 2H, C*H*_2_CH_3_), 0.96 (t, 3H, *J* = 7.4 Hz, CH_2_C*H*_3_), 0.94 (t, 3H, *J* = 7.4 Hz, CH_2_C*H*_3_), 0.92 (t, 3H, *J* = 7.4 Hz, CH_2_C*H*_3_), 0.90 (t, 3H, *J* = 7.4 Hz, CH_2_C*H*_3_); ^13^C-NMR (CD_3_OD, 100 MHz): *δ* 174.6 (C=O), 174.0 (C=O), 173.9 (C=O), 173.5 (C=O), 95.6 (C-1), 79.5 (*C*≡CH), 77.1 (C≡*C*H), 71.8, 71.6, 71.0, 69.5, 69.3, 62.9 (C-6), 56.0 (*C*H_2_-C≡C), 36.9 (*C*H_2_C=O), 36.8 (*C*H_2_C=O), 36.8 (*C*H_2_C=O), 36.8 (*C*H_2_C=O), 19.5 (CH_2_*C*H_3_), 19.4 (*C*H_2_CH_3_), 19.4 (*C*H_2_CH_3_), 19.3 (*C*H_2_CH_3_), 14.1 (CH_2_*C*H_3_), 14.1 (CH_2_*C*H_3_), 14.1 (CH_2_*C*H_3_), 14.1 (CH_2_*C*H_3_); ESIMS: *m/z* 522 [M+Na]^+^.

*2-[2-[2-(2-Propyn-1-yloxy)ethoxy]ethoxy-per-O-butyryl-α-d-glucopyranoside* (**22**). Yield: 92%. ^1^H-NMR (CD_3_OD, 400 MHz): *δ* 5.48 (d, 1H, *J* = 9.9 Hz, C^3^-H), 5.12 (d, 1H, *J* = 3.2 Hz, C^1^-H), 5.10 (t, 1H, *J* = 9.7 Hz, C^4^-H), 4.82 (d, 1H, *J* = 3.1 Hz, C^2^-H), 4.25–4.18 (m, 3H, C^6^-CHa, CH_2_-C≡C), 4.15–4.10 (m, 2H, C^6^-CHb, C^5^-H), 3.68 (m, 6H), 3.65 (m, 6H), 2.85 (s, 1H, C≡CH), 2.35-2.20 (m, 2H, COCH_2_), 2.35–2.20 (m, 2H, COCH_2_), 2.35–2.20 (m, 2H, COCH_2_), 2.35–2.20 (m, 2H, COCH_2_), 1.65–1.56 (m, 2H, C*H*_2_CH_3_), 1.65–1.56 (m, 2H, C*H*_2_CH_3_), 1.65–1.56 (m, 2H, C*H*_2_CH_3_), 1.65–1.56 (m, 2H, C*H*_2_CH_3_), 0.96 (t, 3H, *J* = 3.2 Hz, CH_2_C*H*_3_), 0.94 (t, 3H, *J* = 3.2 Hz, CH_2_C*H*_3_), 0.92 (t, 3H, *J* = 3.2 Hz, CH_2_C*H*_3_), 0.90 (t, 3H, *J* = 3.2 Hz, CH_2_C*H*_3_); ^13^C-NMR (CD_3_OD, 100 MHz): *δ* 174.7 (C=O), 174.0 (C=O), 173.9 (C=O), 173.6 (C=O), 97.1 (C-1), 80.6 (*C*≡CH), 76.0 (C≡*C*H), 72.1, 71.7, 71.6, 71.5, 71.2, 71.2, 70.1, 69.6, 68.7, 62.9 (C-6), 59.0 (*C*H_2_-C≡C), 36.9 (*C*H_2_C=O), 36.8 (*C*H_2_C=O), 36.8 (*C*H_2_C=O), 36.7 (*C*H_2_C=O), 19.4 (*C*H_2_CH_3_), 19.4 (*C*H_2_CH_3_), 19.3 (*C*H_2_CH_3_), 19.3 (*C*H_2_CH_3_), 14.0 (CH_2_*C*H_3_), 14.0 (CH_2_*C*H_3_), 14.0 (CH_2_*C*H_3_), 14.0 (CH_2_*C*H_3_); ESIMS: *m/z* 653 [M+Na]^+^.

*3,6,9,12,15,18-Hexaoxaheneicos-20-yn-1-yl-per-O-butyryl-α-d-glucopyranoside* (**23**). Yield: 92%. ^1^H-NMR (CD_3_OD, 400 MHz): *δ* 5.45 (t, 1H, *J* = 9.8 Hz, C^3^-H), 5.12 (d, 1H, *J* = 3.5 Hz, C^1^-H), 5.07 (t, 1H, *J* = 9.7 Hz, C^4^-H), 4.85 (dd, 1H, *J* = 11.6 Hz, 3.6 Hz, C^2^-H), 4.21–4.19 (m, 3H, C^6^-CHa, CH_2_-C≡C), 4.19–4.13 (m, 2H, C^6^-CHb, C^5^-H), 3.65 (m, 12H), 3.65 (m, 12H), 3.63 (m, 12H), 2.86 (s, 1H, C≡CH), 2.33–2.22 (m, 2H, COCH_2_), 2.33–2.22 (m, 2H, COCH_2_), 2.33–2.22 (m, 2H, COCH_2_), 2.33–2.22 (m, 2H, COCH_2_), 1.65–1.56 (m, 2H, C*H*_2_CH_3_), 1.65–1.56 (m, 2H, C*H*_2_CH_3_), 1.65–1.56 (m, 2H, C*H*_2_CH_3_), 1.65–1.56 (m, 2H, C*H*_2_CH_3_), 0.96 (t, 3H, *J* = 3.8 Hz, CH_2_C*H*_3_), 0.94 (t, 3H, *J* = 3.8 Hz, CH_2_C*H*_3_), 0.92 (t, 3H, *J* = 3.8 Hz, CH_2_C*H*_3_), 0.91 (t, 3H, *J* = 3.8 Hz, CH_2_C*H*_3_); ^13^C-NMR (CD_3_OD, 100 MHz): *δ* 174.7 (C=O), 174.0 (C=O), 173.9 (C=O), 173.6 (C=O), 97.1 (C-1), 80.6 (*C*≡CH), 76.0 (C≡*C*H), 72.1, 71.7, 71.6, 71.6, 71.6, 71.6, 71.4, 71.3, 71.2, 70.1, 69.6, 68.7, 63.0 (C-6), 59.0 (*C*H_2_-C≡C), 36.9 (*C*H_2_C=O), 36.8 (*C*H_2_C=O), 36.7 (*C*H_2_C=O),. 36.7 (*C*H_2_C=O), 19.4 (*C*H_2_CH_3_), 19.3 (*C*H_2_CH_3_), 19.3 (*C*H_2_CH_3_), 19.3 (*C*H_2_CH_3_), 14.0 (CH_2_*C*H_3_), 14.0 (CH_2_*C*H_3_), 14.0 (CH_2_*C*H_3_), 14.0 (CH_2_*C*H_3_); ESIMS: *m/z* 785 [M+Na]^+^.

### 3.4. Click Chemistry-General Procedure

To a solution of a terminal-alkyne **12**–**23** (0.25 mmol) and 4*β*-azido-podophyllotoxin analogue (**7** or **8**, 0.25 mmol) in *t−*BuOH/H_2_O (1:2, 1.0 mL) and THF (1.0 mL) at room temperature were added copper (II) acetate (4.6 mg, 0.025 mmol) and sodium ascorbate (1.0 M in H_2_O, 0.1 mL). The reaction mixture was stirred at room temperature for 31 h until the starting material disappeared as indicated by TLC. Then, the mixture was diluted with water (30 mL) and extracted with ethyl acetate (3 × 20 mL), and the combined organic layer was dried over sodium sulfate. The solvent was evaporated and the residue was purified by column chromatography to afford the cycloaddition product. 

*4β-[4-(α-d-Glucopyranosyloxymethyl)-1,2,3-triazol-1-yl]-4-deoxypodophyllotoxin* (**24**). White amorphous powder, yield 98% (after chromatography with CHCl_3_/CH_3_OH, 9:1); mp 168 °C; [α]_D_^24.1^: +14.2 (c 0.22, CH_3_OH); ^1^H-NMR (CD_3_OD, 500 MHz): *δ* 7.84 (s, 1H, C^5^^''^-H), 6.65 (s, 1H, C^5^-H), 6.58 (s, 1H, C^8^-H), 6.41 (s, 2H, C^2^^'^, C^6^^'^-H), 6.22 (d, 1H, *J* = 4.7 Hz, C^4^-H), 5.93 (d, 2H, *J* = 7.2 Hz, OCH_2_O), 4.90 (d, 1H, *J* = 2.8 Hz, C^1'''^-H), 4.76 (d, 1H, *J* = 4.0 Hz, C^1^-H), 4.66 (s, 2H, C^6''^-CH_2_); 4.63–4.61 (m, 1H), 4.38–4.34 (m, 1H), 3.85–3.82 (m, 2H), 3.80 (s, 2H, C^6'''^-CH_2_), 3.72 (s, 6H, C^3''^, C^5''^-OCH_3_), 3.70 (s, 3H, C^4''^-OCH_3_), 3.68–3.58 (m, 2H), 3.45-3.14 (m, 4H); ^13^C-NMR (CD_3_OD, 125 MHz): *δ* 175.8 (C-12), 153.9 (C-3', C-5'), 150.5 (C-7), 149.2 (C-6), 145.6 (C-4''), 138.2 (C-1'), 136.7 (C-9), 134.7 (C-10), 126.8 (C-4'), 125.9 (C-5''), 111.1 (C-5), 109.8 (C-8), 109.3 (C-2', C-6'), 103.3 (OCH_2_O), 99.7 (C-1'''), 74.9 (C-5'''), 73.9 (C-3'''), 73.3 (C-2'''), 71.7 (C-4'''), 68.9 (C-11), 62.6 (C-6''), 61.5 (C-6'''), 61.1 (4'-OCH_3_), 59.8 (C-2), 56.6 (3', 5'-OCH_3_), 44.8 (C-4), 42.4 (C-1), 38.5 (C-3); ESIMS: *m/z* 680 [M+Na]^+^, HRESIMS: calcd for C_31_H_35_N_3_O_13_H [M+H]^+^ 658.2243, found 658.2223.

*4β-{4-[1-(2,3,4,6-Tetra-O-acetyl-α-d-glucopyranosyloxy)-1,2,3-triazol-1-yl]}-4-deoxypodophyllotoxin* (**25**).White amorphous powder, yield 92% (after chromatography with petroleum ether/acetone, 1:1); mp 137 °C; [α]_D_^24.6^: +16.3 (c 0.28, CH_3_OH); ^1^H-NMR (CD_3_OD, 400 MHz): *δ* 7.87 (s, 1H, C^5^^''^-H), 6.70 (s, 1H, C^5^-H), 6.59 (s, 1H, C^8^-H), 6.41 (s, 2H, C^2^^'^, C^6^^'^-H), 6.25 (d, 1H, *J* = 4.7 Hz, C^4^-H), 5.95 (d, 2H, *J* = 8.3 Hz, OCH_2_O), 5.38 (t, 1H, *J* = 9.7 Hz, C^3'''^-H), 5.18 (d, 1H, *J* = 3.6 Hz, C^1'''^-H), 5.03 (t, 1H, *J* = 8.0 Hz, C^4'''^-H), 4.79–4.67 (m, 5H, C^6''^-CH_2_, C^1^-H, C^2'''^-H, C^5'''^-H), 4.39–4.35 (m, 1H), 4.26–4.20 (m, 1H), 4.11–4.01 (m, 2H), 3.42 (dd, 1H, *J* = 4.0 Hz, 16.0 Hz, C^2^-H), 3.21–3.16 (m, 1H, C^3^-H), 2.04 (s, 3H, COCH_3_), 1.99 (s, 3H, COCH_3_), 1.97 (s, 3H, COCH_3_), 1.95 (s, 3H, COCH_3_); ^13^C-NMR (CD_3_OD, 100 MHz): *δ* 175.8 (C-12), 172.3 (C=O), 171.7 (C=O), 171.5 (C=O), 171.3 (C=O), 153.9 (C-3', C-5'), 150.5 (C-7), 149.3 (C-4''), 144.5 (C-6), 138.3 (C-1'), 136.7 (C-9), 134.7 (C-10), 126.9 (C-4'), 126.4 (C-5''), 111.2 (C-5), 109.9 (C-8), 109.4 (C-2', C-6'), 103.3 (OCH_2_O), 95.6 (C-1'''), 72.2 (C-5'''), 71.2 (C-3'''), 69.8 (C-2'''), 68.9 (C-11), 68.8 (C-4'''), 63.1 (C-6''), 61.3 (C-6'''), 61.1 (4'-OCH_3_), 59.8 (C-2), 56.6 (3', 5'-OCH_3_), 44.9 (C-4), 42.5 (C-1), 38.5 (C-3), 20.7 (CO*C*H_3_), 20.7 (CO*C*H_3_), 20.7 (CO*C*H_3_), 20.7 (CO*C*H_3_); ESIMS: *m/z* 848 [M+Na]^+^, HRESIMS: calcd for C_39_H_43_N_3_O_17_Na [M+Na]^+^ 848.2485, found 848.2472.

*4β-{4-[1-(2,3,4,6-Tetra-O-butyryl-α-d-glucopyranosyloxy)-1,2,3-triazol-1-yl]}-4-deoxypodophyllotoxin* (**26**). White amorphous powder, yield 98% (after chromatography with petroleum ether/acetone, 1:1); mp 101 °C; [α]_D_^25.1^: −41.3 (c 0.23, CH_3_OH); ^1^H-NMR (CD_3_OD, 400 MHz): *δ* 7.74 (s, 1H, C^5^^''^-H), 6.67 (s, 1H, C^5^-H), 6.59 (s, 1H, C^8^-H), 6.41 (s, 2H, C^2^^'^, C^6^^'^-H), 6.24 (d, 1H, *J* = 4.5 Hz, C^4^-H), 5.95 (d, 2H, *J* = 5.6 Hz, OCH_2_O), 5.26 (t, 1H, *J* = 9.4 Hz, C^3'''^-H), 5.08 (t, 1H, *J* = 12.0 Hz, C^4'''^-H), 4.93–4.73 (m, 6H, C^1'''^-H, C^6''^-CH_2_, C^1^-H, C^2'''^-H, C^5'''^-H), 4.33-3.89 (m, 4H), 3.72 (s, 6H, C^3''^, C^5''^-OCH_3_), 3.70 (s, 3H, C^4''^-OCH_3_), 3.40 (dd, 1H, *J* = 4.0 Hz, 16.0 Hz, C^2^-H), 3.14–3.10 (m, 1H, C^3^-H), 2.31–2.13 (m, 2H, COCH_2_), 2.31–2.13 (m, 2H, COCH_2_), 2.31–2.13 (m, 2H, COCH_2_), 2.31–2.13 (m, 2H, COCH_2_), 1.63–1.47 (m, 2H, C*H*_2_CH_3_), 1.63–1.47 (m, 2H, C*H*_2_CH_3_), 1.63–1.47 (m, 2H, C*H*_2_CH_3_), 1.63-1.47 (m, 2H, C*H*_2_CH_3_), 0.92 (t, 3H, *J* = 4.1 Hz, CH_2_C*H*_3_), 0.90 (t, 3H, *J* = 4.1 Hz, CH_2_C*H*_3_), 0.87 (t, 3H, *J* = 4.1 Hz, CH_2_C*H*_3_), 0.83 (t, 3H, *J* = 4.1 Hz, CH_2_C*H*_3_); ^13^C-NMR (CD_3_OD, 100 MHz): *δ* 175.6 (C-12), 174.6 (C=O), 173.8 (C=O), 173.5 (C=O), 173.5 (C=O), 153.9 (C-3', C-5'), 150.5 (C-7), 149.2 (C-4''), 145.3 (C-6), 138.3 (C-1'), 136.6 (C-9), 134.7 (C-10), 126.9 (C-4'), 125.9 (C-5''), 111.2 (C-5), 109.8 (C-8), 109.4 (C-2', C-6'), 103.3 (OCH_2_O), 101.1 (C-1'''), 73.8 (C-5'''), 73.1 (C-3'''), 72.4 (C-2'''), 69.4 (C-4'''), 68.8 (C-11), 63.3 (C-6''), 62.7 (C-6'''), 61.1 (4'-OCH_3_), 59.8 (C-2), 56.6 (3', 5'-OCH_3_), 44.9 (C-4), 42.5 (C-1), 38.5 (C-3), 36.8 (CO*C*H_2_), 36.7 (CO*C*H_2_), 36.7 (CO*C*H_2_), 36.7 (CO*C*H_2_), 19.4 (*C*H_2_CH_3_), 19.3 (*C*H_2_CH_3_), 19.3 (*C*H_2_CH_3_), 19.2 (*C*H_2_CH_3_), 14.0 (CH_2_*C*H_3_), 14.0 (CH_2_*C*H_3_), 14.0 (CH_2_*C*H_3_), 14.0 (CH_2_*C*H_3_); ESIMS: *m/z* 960 [M+Na]^+^, HRESIMS: calcd for C_47_H_59_N_3_O_17_H [M+H]^+^ 938.3917, found 938.3877.

*4β-[4-(α-d-Glucopyranosyloxymethyl)-1,2,3-triazol-1-yl]-4-deoxy-4'-demethylpodophyllotoxin* (**27**). White amorphous powder, yield 98% (after chromatography with CHCl_3_/CH_3_OH, 9:1); mp 227 °C; [α]_D_^24.9^: +1.0 (c 0.30, CH_3_OH); ^1^H-NMR (CD_3_OD, 400 MHz): *δ* 7.87 (s, 1H, C^5^^''^-H), 6.68 (s, 1H, C^5^-H), 6.64 (s, 1H, C^8^-H), 6.38 (s, 2H, C^2^^'^, C^6^^'^-H), 6.26 (d, 1H, *J* = 4.8 Hz, C^4^-H), 5.97 (d, 2H, *J* = 9.7 Hz, OCH_2_O), 4.84–4.42 (m, 6H, C^1'''^-H, C^1^-H, C^6''^-CH_2_, C^11^-CH_2_), 3.83–3.78 (m, 3H, C^3'''^-H, C^6'''^-CH_2_), 3.75 (s, 6H, C^3''^, C^5''^-OCH_3_), 3.67–3.60 (m, 3H), 3.43–3.34 (m, 1H, C^2^-H), 3.26–3.18 (m, 1H, C^3^-H); ^13^C-NMR (CD_3_OD, 100 MHz): *δ* 176.5 (C-12), 150.7 (C-7), 149.6 (C-6), 148.1 (C-3', C-5'), 145.7 (C-4''), 135.9 (C-1'), 135.2 (C-9), 131.5 (C-10), 126.8 (C-4'), 126.2 (C-5''), 111.3 (C-5), 109.2 (C-8), 109.9 (C-2', C-6'), 103.4 (OCH_2_O), 99.8 (C-1'''), 74.9 (C-5'''), 74.0 (C-3'''), 73.4 (C-2'''), 71.7 (C-4'''), 69.2 (C-11), 62.7 (C-6''), 61.5 (C-6'''), 60.1 (C-2), 56.9 (3', 5'-OCH_3_), 44.8 (C-4), 42.8 (C-1), 38.6 (C-3); ESIMS: *m/z* 667 [M+Na]^+^, HRESIMS: calcd for C_30_H_34_N_3_O_13_Na [M+Na]^+^ 667.1984, found 667.1961.

*4β-{4-[1-(2,3,4,6-Tetra-O-acetyl-α-d-glucopyranosyloxy)-1,2,3-triazol-1-yl]}-4-deoxy-4'-demethylpodophyllotoxin* (**28**). White amorphous powder, yield 94% (after chromatography with petroleum ether/acetone, 1:1); mp 128 °C; [α]_D_^24.8^: +27.0 (c 0.14, CH_3_OH); ^1^H-NMR (CD_3_OD, 400 MHz): *δ* 8.24 (s, 1H, C^5^^''^-H), 6.60 (s, 1H, C^5^-H), 6.26 (s, 1H, C^8^-H), 6.58 (s, 2H, C^2^^'^, C^6^^'^-H), 6.02–5.91 (m, 3H, C^4^-H, OCH_2_O), 5.42 (t, 1H, *J* = 9.9 Hz, C^3'''^-H), 5.24 (d, 1H, *J* = 3.5 Hz, C^1'''^-H), 5.04 (t, 1H, *J* = 9.4 Hz, C^4'''^-H), 4.85 (s, 2H, C^6''^-CH_2_), 4.81–4.76 (m, 2H, C^2'''^-H, C^5'''^-H), 4.67 (d, 1H, *J* = 4.2 Hz, C^1^-H), 4.26–4.00 (m, 4H, C^6'''^-CH_2_, C^11^-CH_2_), 3.78 (s, 6H, C^3''^, C^5''^-OCH_3_), 3.57-3.46 (m, 1H, C^3^-H), 3.25 (dd, 1H, *J* = 4.0 Hz, 16.0 Hz, C^2^-H), 2.03 (s, 3H, COCH_3_), 2.00 (s, 3H, COCH_3_), 1.99 (s, 3H, COCH_3_), 1.98 (s, 3H, COCH_3_); ^13^C-NMR (CD_3_OD, 100 MHz): *δ* 176.0 (C-12), 172.3 (C=O), 171.7 (C=O), 171.5 (C=O), 171.2 (C=O), 149.7 (C-7), 149.1(C-6), 148.6 (C-3', C-5'), 144.7 (C-4''), 135.5 (C-1'), 134.3 (C-9), 131.6 (C-10), 129.1 (C-4'), 126.2 (C-5''), 110.9 (C-5), 109.2 (C-2', C-6'), 107.2 (C-8), 103.1 (OCH_2_O), 96.2 (C-1'''), 72.1 (C-5'''), 71.3 (C-3'''), 71.3 (C-11), 69.8 (C-2'''), 68.9 (C-4'''), 63.9 (C-2), 62.9 (C-6''), 61.7 (C-6'''), 56.8 (3', 5'-OCH_3_), 46.6 (C-4), 45.1 (C-1), 39.9 (C-3), 20.6 (CO*C*H_3_), 20.6 (CO*C*H_3_), 20.6 (CO*C*H_3_), 20.5 (CO*C*H_3_); ESIMS: *m/z* 834 [M+Na]^+^, HRESIMS: calcd for C_38_H_41_N_3_O_17_Na [M+H]^+^ 812.2509, found 812.2480.

*4β-{4-[1-(2,3,4,6-Tetra-O-butyryl-α-d-glucopyranosyloxy)-1,2,3-triazol-1-yl]}-4-deoxy-4'-demethylpodophyllotoxin* (**29**). White amorphous powder, yield 96% (after chromatography with petroleum ether/acetone, 1:1); mp 102 °C; [α]_D_^24.7^: +25.9 (c 0.29, CH_3_OH); ^1^H-NMR (CD_3_OD, 400 MHz): *δ* 8.21 (s, 1H, C^5^^''^-H), 6.57 (s, 3H, C^2^^'^, C^6^^'^-H, C^5^-H), 6.18 (s, 1H, C^8^-H), 5.91 (d, 2H, *J* = 6.0 Hz, OCH_2_O), 5.93–5.88 (m, 3H, OCH_2_O, C^4^-H), 5.47 (t, 1H, *J* = 9.7 Hz, C^3'''^-H), 5.25 (d, 1H, *J* = 3.0 Hz, C^1'''^-H), 5.11 (t, 1H, *J* = 9.7 Hz, C^4'''^-H), 4.84 (s, 2H, C^6''^-CH_2_), 4.81 (d, 1H, *J* = 4.0 Hz, C^1^-H), 4.77–4.61 (m, 2H), 4.23–4.03 (m, 4H, C^6'''^-CH_2_, C^11^-CH_2_), 3.55–3.44 (m, 1H, C^3^-H), 3.06 (d, 1H, *J* = 4.0 Hz, 16.0 Hz, C^2^-H), 2.29–2.14 (m, 2H, COCH_2_), 2.29-2.14 (m, 2H, COCH_2_), 2.29–2.14 (m, 2H, COCH_2_), 2.29–2.14 (m, 2H, COCH_2_), 1.62–1.47 (m, 2H, C*H*_2_CH_3_), 1.62–1.47 (m, 2H, C*H*_2_CH_3_), 1.62–1.47 (m, 2H, C*H*_2_CH_3_), 1.62–1.47 (m, 2H, C*H*_2_CH_3_), 0.90 (t, 3H, *J* = 7.8 Hz, CH_2_C*H*_3_), 0.88 (t, 3H, *J* = 7.8 Hz, CH_2_C*H*_3_), 0.86 (t, 3H, *J* = 7.8 Hz, CH_2_C*H*_3_), 0.81 (t, 3H, *J* = 7.8 Hz, CH_2_C*H*_3_); ^13^C-NMR (CD_3_OD, 100 MHz): *δ* 175.8 (C-12), 174.7 (C=O), 174.0 (C=O), 173.9 (C=O), 173.5 (C=O), 149.5 (C-7), 149.0 (C-6), 148.5 (C-3', C-5'), 144.6 (C-4''), 135.4 (C-1'), 134.3 (C-9), 131.7 (C-10), 129.0 (C-4'), 126.2 (C-5''), 111.0 (C-5), 109.2 (C-2', C-6'), 107.3 (C-8), 103.1 (OCH_2_O), 96.3 (C-1'''), 72.0 (C-5'''), 71.1 (C-3'''), 69.5 (C-2'''), 69.1 (C-4'''), 71.3 (C-11), 63.8 (C-2), 62.8 (C-6''), 61.7 (C-6'''), 56.9 (3', 5'-OCH_3_), 46.4 (C-4), 45.0 (C-1), 39.8 (C-3), 36.9 (CO*C*H_2_), 36.9 (CO*C*H_2_), 36.8 (CO*C*H_2_), 36.7 (CO*C*H_2_), 19.4 (*C*H_2_CH_3_), 19.3 (*C*H_2_CH_3_), 19.3 (*C*H_2_CH_3_), 19.3 (*C*H_2_CH_3_), 14.1 (CH_2_*C*H_3_), 14.1 (CH_2_*C*H_3_), 14.1 (CH_2_*C*H_3_), 14.1 (CH_2_*C*H_3_); ESIMS: *m/z* 946 [M+Na]^+^, HRESIMS: calcd for C_46_H_57_N_3_O_17_H [M+H]^+^ 924.3761, found 924.3722.

*4β-{4-[1-(α-d-Glucopyranosyloxymethyl)-3,6,9-trioxadec-10-yl]-1,2,3-triazol-1-yl}-4-deoxypodophyllotoxin* (**30**). White amorphous powder, yield 91% (after chromatography with CHCl_3_/CH_3_OH, 9:1); [α]_D_^25.0^: +0.5 (c 0.15, CH_3_OH); ^1^H-NMR (CD_3_OD, 600 MHz): *δ* 7.84 (s, 1H, C^5^^''^-H), 6.70 (s, 1H, C^5^-H), 6.63 (s, 1H, C^8^-H), 6.41 (s, 2H, C^2^^'^, C^6^^'^-CH), 6.27 (d, 1H, *J* = 3.2 Hz, C^4^-H), 5.98 (d, 2H, *J* = 4.8 Hz, OCH_2_O), 4.82 (d, 1H, *J* = 3.5 Hz, C^1'''^-H), 4.81 (d, 1H, *J* = 4.0 Hz, C^1^-H), 4.62 (s, 2H, C^6''^-CH_2_), 3.79–3.86 (m, 4H), 3.74 (s, 6H, C^3''^, C^5''^-OCH_3_), 3.72 (s, 3H, C^4''^-OCH_3_), 3.69–3.67 (m, 6H), 3.65–3.61 (m, 6H), 3.47–3.44 (m, 2H), 3.38–3.36 (m, 2H), 3.30–3.27 (m, 1H, C^3^-H), 3.17–3.14 (m, 1H, C^2^-H); ^13^C-NMR (CD_3_OD, 150 MHz): *δ* 176.0 (C-12), 154.1 (C-3', C-5'), 150.7 (C-7), 149.5 (C-6), 146.1 (C-4''), 138.4 (C-1'), 136.9 (C-9), 134.9 (C-10), 127.1 (C-4'), 126.1 (C-5''), 111.3 (C-5), 110.0 (C-8), 109.4 (C-2', C-6'), 103.5 (OCH_2_O), 100.5 (C-1'''), 75.3 (C-5'''), 73.8 (C-3'''), 73.8 (C-2'''), 71.9 (C-4'''), 71.6, 71.6, 71.6, 71.5, 71.1 (C-11), 69.1, 68.3, 65.1 (C-6''), 62.8 (C-6'''), 61.2 (4'-OCH_3_), 59.9 (C-2), 56.8 (3', 5'-OCH_3_), 45.1 (C-4), 42.7 (C-1), 38.7 (C-3); ESIMS: *m/z* 812 [M+Na]^+^, HRESIMS: calcd for C_37_H_47_N_3_O_16_Na [M+Na]^+^ 812.2849, found 812.2822.

*4β-{4-[1-(2,3,4,6-Tetra-O-acetyl-α-d-glucopyranosyloxy)-3,6,9-trioxadec-10-yl]-1,2,3-triazol-1-yl}-4-deoxypodophyllotoxin* (**31**). White amorphous powder, yield 94% (after chromatography with petroleum ether/acetone, 1:1); [α]_D_^25.0^: +14.3 (c 0.20, CH_3_OH); ^1^H-NMR (CD_3_OD, 400 MHz): *δ* 8.18 (s, 1H, C^5^^''^-H), 6.62 (s, 2H, C^2^^'^, C^6^^'^-CH), 6.40 (s, 1H, C^5^-H), 6.25 (s, 1H, C^8^-H), 5.97–5.92 (m, 3H, C^4^-H OCH_2_O), 5.43 (t, 1H, *J* = 12.0 Hz, C^3'''^-H), 5.10 (d, 1H, *J* = 3.1 Hz, C^1'''^-H), 5.02 (t, 1H, *J* = 12.0 Hz, C^4'''^-H), 4.84–4.76 (m, 2H), 4.61 (s, 2H, C^6''^-CH_2_), 4.25–4.06 (m, 4H, C^6'''^-CH_2_, C^11^-CH_2_), 3.78 (s, 6H, C^3''^, C^5''^-OCH_3_), 3.72 (s, 3H, C^4''^-OCH_3_), 3.71–3.60 (m, 12H), 3.25 (dd, 1H, *J* = 4.0 Hz, 16.0 Hz, C^2^-H), 3.19–3.14 (m, 1H, C^3^-H), 2.02 (s, 3H, COCH_3_), 1.99 (s, 3H, COCH_3_), 1.98 (s, 3H, COCH_3_), 1.96 (s, 3H, COCH_3_); ^13^C-NMR (CD_3_OD, 100 MHz): *δ* 175.8 (C-12), 172.3 (C=O), 171.6 (C=O), 171.6 (C=O), 171.3 (C=O), 153.9 (C-3', C-5'), 150.5 (C-4''), 149.7 (C-7), 149.3 (C-6), 136.9 (C-1'), 134.7 (C-9), 133.9 (C-10),127.0 (C-4'), 125.6 (C-5''), 110.9 (C-5), 109.9 (C-8), 109.4 (C-2', C-6'), 103.2 (OCH_2_O), 97.1 (C-1'''), 72.1 (C-5'''), 71.7, 71.7, 71.6, 71.6, 71.5, 71.5, 71.2, 71.0, 71.0, 69.9 (C-2'''), 68.7 (C-11), 68.5 (C-4'''), 64.6 (C-6''), 63.2 (C-6'''), 61.1 (4'-OCH_3_), 56.7 (C-2), 56.6 (3', 5'-OCH_3_), 45.2 (C-4), 45.5 (C-1), 39.9 (C-3), 20.7 (CO*C*H_3_), 20.7 (CO*C*H_3_), 20.7 (CO*C*H_3_), 20.7 (CO*C*H_3_); ESIMS: *m/z* 980 [M+Na]^+^, HRESIMS: calcd for C_45_H_55_N_3_O_20_H [M+H]^+^ 958.3452, found 958.3403.

*4β-{4-[1-(2,3,4,6-Tetra-O-butyryl-α-d-glucopyranosyloxy)-3,6,9-trioxadec-10-yl]-1,2,3-triazol-1-yl}-4-deoxypodophyllotoxin* (**32**). White amorphous powder, yield 90% (after chromatography with petroleum ether/acetone, 1:1); [α]_D_^24.0^: +19.3 (c 0.14, CH_3_OH); ^1^H-NMR (CD_3_OD, 400 MHz): *δ* 8.17 (s, 1H, C^5^^''^-H), 6.61 (s, 2H, C^2^^'^, C^6^^'^-H), 6.40 (s, 1H, C^5^-H), 6.25 (s, 1H, C^8^-H), 5.97–5.90 (m, 3H, C^4^-H, OCH_2_O), 5.48 (t, 1H, *J* = 12.0 Hz, C^3'''^-H), 5.11–5.05 (m, 2H, C^1'''^-H, C^4'''^-H), 4.87–4.83 (m, 2H), 4.76 (d, 1H, *J* = 4.0 Hz, C^1^-H), 4.60 (s, 2H, C^6''^-CH_2_), 4.23–4.08 (m, 4H, C^6'''^-CH_2_, C^11^-CH_2_), 3.71 (s, 6H, C^3''^, C^5''^-OCH_3_), 3.70 (s, 3H, C^4''^-OCH_3_), 3.70–3.59 (m, 12H), 3.22–3.12 (m, 2H, C^2^-H, C^3^-H), 2.31–2.19 (m, 2H, COCH_2_), 2.31–2.19 (m, 2H, COCH_2_), 2.31–2.19 (m, 2H, COCH_2_), 2.31–2.19 (m, 2H, COCH_2_), 1.63–1.53 (m, 2H, C*H*_2_CH_3_), 1.63–1.53 (m, 2H, C*H*_2_CH_3_), 1.63-1.53 (m, 2H, C*H*_2_CH_3_), 1.63-1.53 (m, 2H, C*H*_2_CH_3_), 0.92 (t, 3H, *J* = 4.0 Hz, CH_2_C*H*_3_), 0.90 (t, 3H, *J* = 4.0 Hz, CH_2_C*H*_3_), 0.88 (t, 3H, *J* = 4.0 Hz, CH_2_C*H*_3_), 0.86 (t, 3H, *J* = 4.0 Hz, CH_2_C*H*_3_); ^13^C-NMR (CD_3_OD, 100 MHz): *δ* 175.7 (C-12), 174.7 (C=O), 174.1 (C=O), 174.0 (C=O), 173.6 (C=O), 153.9 (C-3', C-5'), 150.5 (C-4''), 149.6 (C-7), 146.2 (C-6), 137.9 (C-9), 136.9 (C-10), 133.9 (C-1'), 127.0 (C-4'), 125.6 (C-5''), 110.9 (C-5), 109.8 (C-8), 109.4 (C-2', C-6'), 103.2 (OCH_2_O), 97.1 (C-1'''), 72.1 (C-5'''), 71.7, 71.7, 71.6, 71.6, 71.6, 71.2 (C-3'''), 69.6 (C-2'''), 68.7 (C-4'''), 68.6 (C-11), 65.2 (C-6''), 63.0 (C-6'''), 61.1 (4'-OCH_3_), 56.7 (C-2), 56.7 (3', 5'-OCH_3_), 45.2 (C-4), 42.5 (C-1), 39.9 (C-3), 36.8 (CO*C*H_2_), 36.8 (CO*C*H_2_), 36.8 (CO*C*H_2_), 36.8 (CO*C*H_2_), 19.4 (*C*H_2_CH_3_), 19.3 (*C*H_2_CH_3_), 19.3 (*C*H_2_CH_3_), 19.3 (*C*H_2_CH_3_), 14.1 (CH_2_*C*H_3_), 14.1 (CH_2_*C*H_3_), 14.1 (CH_2_*C*H_3_), 14.1 (CH_2_*C*H_3_); ESIMS: *m/z* 1092 [M+Na]^+^, HRESIMS: calcd for C_53_H_71_N_3_O_20_H [M+H]^+^ 1070.4704, found 1070.4658.

*4β-{4-[1-(α-d-Glucopyranosyloxymethyl)-3,6,9-trioxadec-10-yl]-1,2,3-triazol-1-yl}-4-deoxy-4'-demethylpodophyllotoxin* (**33**). White amorphous powder, yield 93% (after chromatography with CHCl_3_/CH_3_OH, 9:1); [α]_D_^25.3^: −37.2 (c 0.11, CH_3_OH); ^1^H-NMR (CD_3_OD, 600 MHz): *δ* 7.83 (s, 1H, C^5''^-H), 6.69 (s, 1H, C^5^-H), 6.66 (s, 1H, C^8^-H), 6.38 (s, 2H, C^2^^'^, C^6^^'^-H), 6.27 (d, 1H, *J* = 3.2 Hz, C^4^-H), 5.99 (d, 2H, *J* = 5.6 Hz, OCH_2_O), 4.82 (d, 1H, *J* = 4.0 Hz, C^1'''^-H), 4.78 (d, 1H, *J* = 4.0 Hz, C^1^-H), 4.63 (s, 2H, C^6''^-CH_2_), 3.86–3.79 (m, 4H, C^6'''^-CH_2_, C^11^-CH_2_), 3.75 (s, 6H, C^3''^, C^5''^-OCH_3_), 3.69–3.61 (m, 12H), 3.44–3.40 (m, 2H), 3.38–3.36 (m, 2H), 3.30–3.26 (m, 2H), 3.18–3.14 (m, 2H, C^2^-H, C^3^-H); ^13^C-NMR (CD_3_OD, 150 MHz): *δ* 176.2 (C-12), 150.7 (C-7), 149.4 (C-4''), 148.8 (C-3', C-5'), 146.1 (C-6), 136.1 (C-1'), 135.3 (C-9), 131.5 (C-10), 127.1 (C-4'), 126.1 (C-5''), 111.4 (C-5), 109.9 (C-8), 109.4 (C-2', C-6'), 103.4 (OCH_2_O), 100.5 (C-1'''), 75.3 (C-5'''), 73.8 (C-3'''), 73.8 (C-2'''), 72.0 (C-4'''), 71.7, 71.6, 71.6, 71.5 (C-11), 71.1, 69.1, 68.3, 65.1 (C-6''), 62.8 (C-6'''), 60.0 (C-2), 56.9 (3', 5'-OCH_3_), 44.9 (C-4), 42.8 (C-1), 38.7 (C-3); ESIMS: *m/z* 798 [M+Na]^+^, HRESIMS: calcd for C_36_H_45_N_3_O_16_Na [M+Na]^+^ 798.2692, found 798.2669.

*4β-{4-[1-(2,3,4,6-Tetra-O-acetyl-α-d-glucopyranosyloxy)-3,6,9-trioxadec-10-yl]-1,2,3-triazol-1-yl}-4-deoxy-4'-demethylpodophyllotoxin* (**34**). White amorphous powder, yield 94% (after chromatography with petroleum ether/acetone, 1:1); [α]_D_^23.8^: +14.4 (c 0.23, CH_3_OH); ^1^H-NMR (CD_3_OD, 400 MHz): *δ* 8.18 (s, 1H, C^5''^-H), 6.61 (s, 1H, C^5^-H), 6.58 (s, 2H, C^2^^'^, C^6^^'^-H), 6.25 (s, 1H, C^8^-H), 6.03–5.93 (m, 3H, C^4^-H, OCH_2_O), 5.42 (d, 1H, *J* = 8.0 Hz, C^3'''^-H), 5.10 (d, 1H, *J* = 2.8 Hz, C^1'''^-H), 5.02 (t, 1H, *J* = 8.0 Hz, C^4'''^-H), 4.83–4.81 (m, 2H), 4.70–4.67 (m, 3H, C^6''^-CH_2_, C^1^-H), 4.25–4.07 (m, 4H, C^6'''^-CH_2_, C^11^-CH_2_), 3.79 (s, 6H, C^3''^, C^5''^-OCH_3_), 3.72–3.63 (m, 12H), 3.57–3.45 (m, 1H, C^3^-H), 3.26 (dd, 1H, *J* = 4.0 Hz, 16.0 Hz, C^2^-H), 2.02 (s, 3H, COCH_3_), 2.01 (s, 3H, COCH_3_), 1.99 (s, 3H, COCH_3_), 1.96 (s, 3H, COCH_3_); ^13^C-NMR (CD_3_OD, 100 MHz): *δ* 176.0 (C-12), 172.4 (C=O), 171.8 (C=O), 171.7 (C=O), 171.7 (C=O), 149.7 (C-7), 149.1 (C-6), 148.6 (C-3', C-5'), 145.8 (C-4''), 135.6 (C-1'), 134.3 (C-9), 131.6 (C-10), 129.2 (C-4'), 125.6 (C-5''), 110.9 (C-5), 109.2 (C-2', C-6'), 107.2 (C-8), 103.1 (OCH_2_O), 97.1 (C-1'''), 72.1 (C-5'''), 71.7, 71.6, 71.6,71.5 (C-3'''), 71.2, 71.0, 70.0 (C-2'''), 68.7 (C-11), 68.5 (C-4'''), 65.1 (C-6''), 63.9 (C-2), 63.2 (C-6'''), 56.8 (3', 5'-OCH_3_), 46.6 (C-4), 45.1 (C-1), 39.9 (C-3), 20.7 (CO*C*H_3_), 20.6 (CO*C*H_3_), 20.6 (CO*C*H_3_), 20.6 (CO*C*H_3_); ESIMS: *m/z* 966 [M+Na]^+^, HRESIMS: calcd for C_44_H_53_N_3_O_20_H [M+H]^+^ 944.3295, found 944.3249.

*4β-{4-[1-(2,3,4,6-Tetra-O-butyryl-α-d-glucopyranosyloxy)-3,6,9-trioxadec-10-yl]-1,2,3-triazol-1-yl}-4-deoxy-4'-demethylpodophyllotoxin* (**35**). White amorphous powder, yield 92% (after chromatography with petroleum ether/acetone, 1:1); [α]_D_^25.0^: +22.4 (c 0.24, CH_3_OH); ^1^H-NMR (CD_3_OD, 400 MHz): *δ* 8.18 (s, 1H, C^5''^-H), 6.61 (s, 1H, C^5^-H), 6.59 (s, 2H, C^2'^-H, C^6'^-H), 6.26 (s, 1H, C^8^-H), 6.04–5.94 (m, 3H, C^4^-H, OCH_2_O), 5.47 (t, 1H, *J* = 12.0 Hz, C^3'''^-H), 5.11 (d, 2H, *J* = 3.7 Hz, C^1'''^-H), 5.06 (t, 1H, *J* = 8.0 Hz, C^4'''^-H), 4.85–4.81 (m, 2H), 4.71–4.69 (m, 3H, C^6''^-CH_2_, C^1^-H), 4.23–4.09 (m, 4H, C^6'''^-CH_2_, C^11^-CH_2_), 3.80 (s, 6H, C^3''^, C^5''^-OCH_3_), 3.72–3.64 (m, 12H), 3.55–3.49 (m, 1H, C^3^-H), 3.28–3.26 (m, 1H, C^2^-H), 2.32–2.19 (m, 2H, COCH_2_), 2.32–2.19 (m, 2H, COCH_2_), 2.32–2.19 (m, 2H, COCH_2_), 2.32–2.19 (m, 2H, COCH_2_), 1.62–1.53 (m, 2H, C*H*_2_CH_3_), 1.62–1.53 (m, 2H, C*H*_2_CH_3_), 1.62–1.53 (m, 2H, C*H*_2_CH_3_), 1.62–1.53 (m, 2H, C*H*_2_CH_3_), 0.93 (t, 3H, *J* = 4.0 Hz, CH_2_C*H*_3_), 0.91 (t, 3H, *J* = 4.0 Hz, CH_2_C*H*_3_), 0.89 (t, 3H, *J* = 4.0 Hz, CH_2_C*H*_3_), 0.87 (t, 3H, *J* = 4.0 Hz, CH_2_C*H*_3_); ^13^C-NMR (CD_3_OD, 100 MHz): *δ* 176.0 (C-12), 174.8 (C=O), 174.1 (C=O), 174.1 (C=O), 173.6 (C=O), 149.7 (C-7), 149.1 (C-6), 148.6 (C-3', C-5'), 134.3 (C-1'), 131.5 (C-9), 131.5 (C-10), 127.2 (C-4'), 125.5 (C-5''), 110.9 (C-5), 109.2 (C-2', C-6'), 107.2 (C-8), 103.1 (OCH_2_O), 97.1 (C-1'''), 72.1 (C-5'''), 71.7, 71.6, 71.6, 71.3, 71.2 (C-3'''), 71.0, 69.6 (C-2'''), 68.7 (C-4'''), 68.7 (C-11), 65.1 (C-6''), 63.9 (C-2), 62.9 (C-6'''), 56.8 (3', 5'-OCH_3_), 46.6 (C-4), 45.2 (C-1), 39.9 (C-3), 36.9 (CO*C*H_2_), 36.8 (CO*C*H_2_), 36.7 (CO*C*H_2_), 36.7 (CO*C*H_2_), 19.4 (*C*H_2_CH_3_), 19.3 (*C*H_2_CH_3_), 19.3 (*C*H_2_CH_3_), 19.2 (*C*H_2_CH_3_), 14.0 (CH_2_*C*H_3_), 14.0 (CH_2_*C*H_3_), 14.0 (CH_2_*C*H_3_), 14.0 (CH_2_*C*H_3_); ESIMS: *m/z* 1078 [M+Na]^+^, HRESIMS: calcd for C_52_H_69_N_3_O_20_H [M+H]^+^ 1056.4547, found 1056.4484.

*4β-{4-[1-(α-d-Glucopyranosyloxymethyl)-3,6,9,12,15,18-hexaoxanonadec-19-yl]-1,2,3-triazol-1-yl}-4-deoxypodophyllotoxin* (**36**). White amorphous powder, yield 91% (after chromatography with CHCl_3_/CH_3_OH, 9:1); [α]_D_^24.3^: −2.5 (c 0.17, CH_3_OH); ^1^H-NMR (CD_3_OD, 400 MHz): *δ* 7.82 (s, 1H, C^5''^-H), 6.69 (s, 1H, C^5^-H), 6.64 (s, 1H, C^8^-H), 6.41 (s, 2H, C^2'^, C^6'^-H), 6.26 (d, 1H, *J* = 4.8 Hz, C^4^-H), 5.98 (d, 2H, *J* = 5.5 Hz, OCH_2_O), 4.82–4.80 (m, 2H, C^1'''^-H, C^1^-H), 4.62 (s, 2H, C^6''^-CH_2_), 3.87–3.78 (m, 4H, C^6'''^-CH_2_, C^11^-CH_2_), 3.74 (s, 6H, C^3''^, C^5''^-OCH_3_), 3.72 (s, 3H, C^4''^-OCH_3_), 3.68–3.60 (m, 24H), 3.47–3.42 (m, 2H), 3.38–3.35 (m, 2H), 3.28–3.25 (m, 1H), 3.18–3.13 (m, 1H); ^13^C-NMR (CD_3_OD, 100 MHz): *δ* 175.8 (C-12), 153.9 (C-3', C-5'), 150.6 (C-7), 149.3 (C-6), 148.1 (C-4''), 138.3 (C-1'), 136.7 (C-9), 134.7 (C-10), 126.9 (C-4'), 125.9 (C-5''), 111.2 (C-5), 109.8 (C-8), 109.3 (C-2', C-6'), 103.3 (OCH_2_O), 100.3 (C-1'''), 75.2 (C-5'''), 73.7 (C-3'''), 73.7 (C-2'''), 71.8 (C-4'''), 71.5, 71.5, 71.5, 71.3, 70.9 (C-11), 68.9, 68.1, 65.0 (C-6''), 62.7 (C-6'''), 61.1 (4'-OCH_3_), 59.8 (C-2), 56.7 (3', 5'-OCH_3_), 44.9 (C-4), 42.5 (C-1), 38.6 (C-3); ESIMS: *m/z* 944 [M+Na]^+^, HRESIMS: calcd for C_43_H_59_N_3_O_19_Na [M+Na]^+^ 944.3635, found 944.3622.

*4β-{4-[1-(2,3,4,6-Tetra-O-acetyl-α-d-glucopyranosyloxy)-3,6,9,12,15,18-hexaoxanonadec-19-yl]-1,2,3-triazol-1-yl}-4-deoxypodophyllotoxin* (**37**). White amorphous powder, yield 91% (after chromatography with petroleum ether/acetone, 1:1); [α]_D_^23.9^: −24.9 (c 0.22, CH_3_OH); ^1^H-NMR (CD_3_OD, 400 MHz): *δ* 8.21 (s, 1H, C^5^^′′^-H), 6.62, (s, 2H, C^2^^′^, C^6^^′^-H), 6.60 (s, 1H, C^5^-H), 6.25 (s, 1H, C^8^-H), 6.05–5.94 (m, 3H, C^4^-H, OCH_2_O), 5.43 (t, 1H, *J* = 12.0 Hz, C^3'''^-H), 5.11 (d, 1H, *J* = 3.5 Hz, C^1'''^-H), 5.02 (t, 1H, *J* = 12.0 Hz, C^4'''^-H), 4.85–4.80 (m, 3H), 4.70 (s, 2H, C^6''^-CH_2_), 4.26–4.03 (m, 4H, C^6'''^-CH_2_, C^11^-CH_2_), 3.79 (s, 6H, C^3''^, C^5''^-OCH_3_), 3.73 (s, 3H, C^4''^-OCH_3_), 3.65–3.59 (m, 24H), 3.55-3.45 (m, 2H, C^2^-H, C^3^-H), 2.04 (s, 3H, COCH_3_), 2.03 (s, 3H, COCH_3_), 2.00 (s, 3H, COCH_3_), 1.97 (s, 3H, COCH_3_); ^13^C-NMR (CD_3_OD, 100 MHz): *δ* 175.8 (C-12), 172.3 (C=O), 171.7 (C=O), 171.7 (C=O), 171.3 (C=O), 153.9 (C-3', C-5'), 153.9 (C-7), 149.7 (C-6), 149.2 (C-4''), 137.9 (C-1'), 137.0 (C-9), 133.9 (C-10), 129.3 (C-4'), 125.7 (C-5''), 110.9 (C-5), 109.3 (C-2', C-6'), 107.2 (C-8), 103.2 (OCH_2_O), 97.1 (C-1'''), 72.1 (C-5'''), 71.7, 71.6, 71.5, 71.5, 71.2 (C-3'''), 71.0 (C-2'''), 70.0, 68.7 (C-4'''), 68.5 (C-11), 65.1 (C-6''), 63.8 (C-2), 63.2 (C-6'''), 61.1 (4'-OCH_3_), 56.7 (3', 5'-OCH_3_), 45.5 (C-4), 45.3 (C-1), 39.9 (C-3), 20.7 (CO*C*H_3_), 20.6 (CO*C*H_3_), 20.6 (CO*C*H_3_), 20.6 (CO*C*H_3_); ESIMS: *m/z* 1090 [M+H]^+^, HRESIMS: calcd for C_51_H_67_N_3_O_23_H [M+H]^+^ 1090.4238, found 1090.4177.

*4β-{4-[1-(2,3,4,6-Tetra-O-butyryl-α-d-glucopyranosyloxy)-3,6,9,12,15,18-hexaoxanonadec-19-yl]-1,2,3-triazol-1-yl}-4-deoxypodophyllotoxin* (**38**). White amorphous powder, yield 91% (after chromatography with petroleum ether/acetone, 1:1); [α]_D_^23.7^: +17.4 (c 0.20, CH_3_OH); ^1^H-NMR (CD_3_OD, 400 MHz): *δ* 8.20 (s, 1H, C^5''^-H), 6.62 (s, 2H, C^2'^, C^6'^-H), 6.59 (s, 1H, C^5^-H), 6.25 (s, 1H, C^8^-H), 6.04–5.93 (m, 3H, C^4^-H, OCH_2_O), 5.48 (t, 1H, *J* = 8.0 Hz, C^3'''^-H), 5.11–5.04 (m, 2H, C^1'''^-H, C^4'''^-H), 4.85–4.81 (m, 2H), 4.72–4.70 (m, 3H, C^6''^-CH_2_, C^1^-H), 4.23–4.09 (m, 4H, C^6'''^-CH_2_, C^11^-CH_2_), 3.79 (s, 6H, C^3''^, C^5''^-OCH_3_), 3.72 (s, 3H, C^4''^-OCH_3_), 3.65–3.61 (m, 24H), 3.27–3.26 (m, 1H), 3.18–3.13 (m, 1H), 2.33–2.20 (m, 2H, COCH_2_), 2.33–2.20 (m, 2H, COCH_2_), 2.33–2.20 (m, 2H, COCH_2_), 2.33-2.20 (m, 2H, COCH_2_), 1.65–1.53 (m, 2H, C*H*_2_CH_3_), 1.65–1.53 (m, 2H, C*H*_2_CH_3_), 1.65–1.53 (m, 2H, C*H*_2_CH_3_), 1.65–1.53 (m, 2H, C*H*_2_CH_3_), 0.94 (t, 3H, *J* = 4.0 Hz, CH_2_C*H*_3_), 0.91 (t, 3H, *J* = 4.0 Hz, CH_2_C*H*_3_), 0.90 (t, 3H, *J* = 4.0 Hz, CH_2_C*H*_3_), 0.88 (t, 3H, *J* = 4.0 Hz, CH_2_C*H*_3_); ^13^C-NMR (CD_3_OD, 100 MHz): *δ* 175.8 (C-12), 174.7 (C=O), 174.1 (C=O), 174.0 (C=O), 173.6 (C=O), 153.9 (C-3', C-5'), 149.7 (C-7), 149.2 (C-6), 146.3 (C-4''), 137.9 (C-1'), 136.9 (C-9), 133.9 (C-10), 129.3 (C-4'), 125.8 (C-5''), 110.9 (C-5), 109.4 (C-2', C-6'), 107.3 (C-8), 103.2 (OCH_2_O), 97.1 (C-1'''), 72.1 (C-5'''), 71.7, 71.6, 71.5, 71.2, 71.0 (C-3'''), 69.6 (C-2'''), 68.7 (C-11), 68.6 (C-4'''), 65.2 (C-6''), 63.8 (C-2), 63.0 (C-6'''), 61.1 (4'-OCH_3_), 56.7 (3', 5'-OCH_3_), 46.5 (C-4), 45.2 (C-1), 39.9 (C-3), 36.9 (CO*C*H_2_), 36.8 (CO*C*H_2_), 36.7 (CO*C*H_2_), 36.7 (CO*C*H_2_), 19.4 (*C*H_2_CH_3_), 19.3 (*C*H_2_CH_3_), 19.3 (*C*H_2_CH_3_), 19.3 (*C*H_2_CH_3_), 14.0 (CH_2_*C*H_3_), 14.0 (CH_2_*C*H_3_), 14.0 (CH_2_*C*H_3_), 14.0 (CH_2_*C*H_3_); ESIMS: *m/z* 1224 [M+Na]^+^, HRESIMS: calcd for C_59_H_83_N_3_O_23_H [M+H]^+^ 1202.5490, found 1202.5423.

*4β-{4-[1-(α-d-Glucopyranosyloxymethyl)-3,6,9,12,15,18-hexaoxanonadec-19-yl]-1,2,3-triazol-1-yl}-4-deoxy-4'-demethylpodophyllotoxin* (**39**). White amorphous powder, yield 93% (after chromatography with CHCl_3_/CH_3_OH, 9:1); mp 158 °C; [α]_D_^25.4^: −4.0 (c 0.11, CH_3_OH); ^1^H-NMR (CD_3_OD, 600 MHz): *δ* 7.82 (s, 1H, C^5^^''^-H), 6.69 (s, 1H, C^5^-H), 6.66 (s, 1H, C^8^-H), 6.38 (s, 2H, C^2^^'^, C^6^^'^-H), 6.27 (d, 1H, *J* = 3.6 Hz, C^4^-H), 5.98 (d, 2H, *J* = 5.6 Hz, OCH_2_O), 4.82 (d, 1H, *J* = 2.4 Hz, C^1'''^-H), 4.78 (d, 1H, *J* = 3.6 Hz, C^1^-H), 4.64 (s, 2H, C^6''^-CH_2_), 3.85–3.80 (m, 4H, C^6'''^-CH_2_, C^11^-CH_2_), 3.75 (s, 6H, C^3''^, C^5''^-OCH_3_), 3.69–3.60 (m, 24H), 3.42–3.40 (m, 2H), 3.38–3.36 (m, 1H), 3.18–3.14 (m ,2H, C^2^-H, C^3^-H); ^13^C-NMR (CD_3_OD, 150 MHz): *δ* 176.2 (C-12), 150.7 (C-7), 149.4 (C-6), 148.8 (C-3', C-5'), 135.2 (C-4''), 132.5 (C-1'), 131.5 (C-9), 130.0 (C-10), 127.1 (C-4'), 126.1 (C-5''), 111.4 (C-5), 109.9 (C-8), 109.4 (C-2', C-6'), 103.4 (OCH_2_O), 100.4 (C-1'''), 75.3 (C-5'''), 73.9 (C-3'''), 73.9 (C-2'''), 71.9 (C-4'''), 71.7, 71.7, 71.6, 71.5, 71.1, 70.9 (C-11), 69.2, 68.2, 65.1 (C-6''), 62.3 (C-6'''), 60.0 (C-2), 56.9 (3', 5'-OCH_3_), 44.9 (C-4), 42.9 (C-1), 68.7 (C-3); ESIMS: *m/z* 930 [M+Na]^+^, HRESIMS: calcd for C_42_H_57_N_3_O_19_Na [M+Na]^+^ 930.3478, found 930.3462.

*4β-{4-[1-(2,3,4,6-Tetra-O-acetyl-α-d-glucopyranosyloxy)-3,6,9,12,15,18-hexaoxanonadec-19-yl]-1,2,3-triazol-1-yl}-4-deoxy-4'-demethylpodophyllotoxin* (**40**). White amorphous powder, yield 90% (after chromatography with petroleum ether/acetone, 1:1); [α]_D_^25.0^: +25.1 (c 0.23, CH_3_OH); ^1^H-NMR (CD_3_OD, 400 MHz): *δ* 8.18 (s, 1H, C^5''^-H), 6.60 (s, 1H, C^5^-H), 3.58 (s, 2H, C^2'^, C^6'^-H), 6.23 (s, 1H, C^8^-H), 6.01–5.92 (m, 3H, C^4^-H, OCH_2_O), 5.43 (t, 1H, *J* = 12.0 Hz, C^3'''^-H), 5.11 (d, 1H, *J* = 4.0 Hz, C^1'''^-H), 5.02 (t, 1H, *J* = 12.0 Hz, C^4'''^-H), 4.69 (s, 2H, C^6''^-CH_2_), 4.65 (d, 1H, *J* = 4.0 Hz, C^1^-H), 4.30–4.07 (m, 4H, C^6'''^-CH_2_, C^11^-CH_2_), 3.78 (s, 6H, C^3''^, C^5''^-OCH_3_), 3.69–3.59 (m, 24H), 3.44–3.41 (m, 1H, C^3^-H), 3.21 (dd, 1H, *J* = 4.0 Hz, 12.0 Hz, C^4^-H), 2.04 (s, 3H, COCH_3_), 2.03 (s, 3H, COCH_3_), 2.00 (s, 3H, COCH_3_), 1.97 (s, 3H, COCH_3_); ^13^C-NMR (CD_3_OD, 100 MHz): *δ* 174.4 (C-12), 170.8 (C=O), 170.2 (C=O), 170.2 (C=O), 169.8 (C=O), 148.1 (C-7), 147.6 (C-6), 147.1 (C-3', C-5'), 144.6 (C-4''), 134.0 (C-1'), 132.7 (C-9), 130.1 (C-10), 128.3 (C-4'), 127.7 (C-5''), 109.4 (C-5), 107.6 (C-2', C-6'), 105.7 (C-8), 101.6 (OCH_2_O), 95.5 (C-1'''), 72.1, 71.6, 71.6, 71.5, 71.2, 71.0 (C-5'''), 70.0 (C-3'''), 68.4 (C-2'''), 67.2 (C-11), 66.9 (C-4'''), 63.5 (C-6''), 62.3 (C-2), 61.6 (C-6'''), 55.3 (3', 5'-OCH_3_), 45.0 (C-4), 43.6 (C-1), 38.3 (C-3), 20.7 (CO*C*H_3_), 20.7 (CO*C*H_3_), 20.7 (CO*C*H_3_), 20.7 (CO*C*H_3_); ESIMS: *m/z* 1098 [M+Na]^+^, HRESIMS: calcd for C_50_H_65_N_3_O_23_H [M+H]^+^ 1076.4082, found 1076.4031.

*4β-{4-[1-(2,3,4,6-Tetra-O-butyryl-α-d-glucopyranosyloxy)-3,6,9,12,15,18-hexaoxanonadec-19-yl]-1,2,3-triazol-1-yl}-4-deoxy-4'-demethylpodophyllotoxin* (**41**). White amorphous powder, yield 97% (after chromatography with petroleum ether/acetone, 1:1); [α]_D_^24.6^: −3.1 (c 0.29, CH_3_OH); ^1^H-NMR (CD_3_OD, 400 MHz): *δ* 8.18 (s, 1H, C^5''^-H), 6.62 (s, 1H, C^5^-H), 6.59 (s, 2H, C^2'^, C^6'^-H), 6.25 (s, 1H, C^8^-H), 5.95–5.84 (m, 3H, C^4^-H, OCH_2_O), 5.37 (t, 1H, *J* = 8.0 Hz, C^3'''^-H), 5.02 (d, 2H, *J* = 4.0 Hz, C^1'''^-H), 4.97 (t, 1H, *J* = 12.0 Hz, C^4'''^-H), 4.61–4.59 (m, 3H, C^6''^-CH_2_, C^1^-H), 4.13–4.00 (m, 4H, C^6'''^-CH_2_, C^11^-CH_2_), 3.80 (s, 6H, C^3''^, C^5''^-OCH_3_), 3.53–3.51 (m, 24H), 3.45–3.39 (m, 2H, C^2^-H, C^3^-H), 2.24–2.10 (m, 8H), 1.56–1.43 (m, 8H), 0.85 (t, 3H, *J* = 4.0 Hz, CH_2_C*H*_3_), 0.82 (t, 3H, *J* = 4.0 Hz, CH_2_C*H*_3_), 0.80 (t, 3H, *J* = 4.0 Hz, CH_2_C*H*_3_), 0.79 (t, 3H, *J* = 4.0 Hz, CH_2_C*H*_3_); ^13^C-NMR (CD_3_OD, 100 MHz): *δ* 176.0 (C-12), 174.8 (C=O), 174.0 (C=O), 174.0 (C=O), 173.6 (C=O), 149.7 (C-7), 149.1 (C-6), 148.6 (C-3', C-5'), 146.2 (C-4''), 135.6 (C-1'), 134.3 (C-9), 131.6 (C-10), 129.2 (C-4'), 125.6 (C-5''), 110.0 (C-5), 109.1 (C-2', C-6'), 107.1 (C-8), 103.0 (OCH_2_O), 97.1 (C-1'''), 72.1 (C-5'''), 71.7, 71.6, 71.5, 71.2 (C-3'''), 69.6 (C-2'''), 68.6 (C-4'''), 68.6 (C-11), 65.1 (C-6''), 63.8 (C-2), 62.9 (C-6'''), 56.8 (3', 5'-OCH_3_), 46.7 (C-4), 45.1 (C-1), 39.9 (C-3), 36.8 (CO*C*H_2_), 36.7 (CO*C*H_2_), 36.7 (CO*C*H_2_), 36.7 (CO*C*H_2_), 19.4 (*C*H_2_CH_3_), 19.3 (*C*H_2_CH_3_), 19.3 (*C*H_2_CH_3_), 19.3 (*C*H_2_CH_3_), 14.0 (CH_2_*C*H_3_), 14.0 (CH_2_*C*H_3_), 14.0 (CH_2_*C*H_3_), 14.0 (CH_2_*C*H_3_); ESIMS: *m/z* 1210 [M+Na]^+^, HRESIMS: calcd for C_58_H_81_N_3_O_23_H [M+H]^+^ 1188.5334, found 1188.5298.

*4β-[4-(α-d-Glucopyranosyloxymethyl)-1,2,3-triazol-1-yl]-4-deoxy-4'-demethylpodophyllotoxin* (**42**). White amorphous powder, yield 97% (after chromatography with CHCl_3_/CH_3_OH, 9:1); mp 148 °C;^1^H-NMR (CD_3_OD, 400 MHz): *δ* 8.22 (s, 1H, C^5''^-H), 6.61 (s, 1H, C^5^-H), 6.59 (s, 2H, C^2'^, C^6'^-H), 6.25 (s, 1H, C^8^-H), 6.03–5.93 (m, 3H, C^4^-H, OCH_2_O), 5.03 (d, 1H, *J* = 12.0 Hz, C^1'''^-H), 4.85 (s, 2H, C^6''^-CH_2_), 4.70 (d, 1H, *J* = 4.3 Hz, C^1^-H), 4.43 (d, 1H, *J* = 4.0 Hz), 4.24–4.16 (m, 2H, C^6'''^-CH_2_), 3.91 (d, 1H, *J* = 12.0 Hz), 3.79 (s, 6H, C^3''^, C^5''^-OCH_3_), 3.68 (dd, 1H, *J* = 4.0 Hz, 12.0 Hz, C^2^-H), 3.57–3.46 (m, 1H, C^3^-H), 3.39–3.35 (m, 2H), 3.27–3.21 (m, 2H); ^13^C-NMR (CD_3_OD, 100 MHz): *δ* 176.1 (C-12), 149.7 (C-7), 149.2 (C-6), 148.6 (C-3', C-5'), 145.9 (C-4''), 135.6 (C-1'), 134.2 (C-9), 131.6 (C-10), 129.2 (C-4'), 125.8 (C-5''), 110.9 (C-5), 109.2 (C-2', C-6'), 107.2 (C-8), 103.8 (C-1'''), 103.1 (OCH_2_O), 78.1 (C-5'''), 77.9 (C-3'''), 75.0 (C-2'''), 71.6 (C-4'''), 71.3 (C-11), 63.9 (C-2), 63.2 (C-6''), 62.7 (C-6'''), 56.8 (3', 5'-OCH_3_), 46.6 (C-4), 45.1 (C-1), 39.9 (C-3); ESIMS: *m/z* 666 [M+Na]^+^, HRESIMS: calcd for C_30_H_33_N_3_O_13_Na [M+Na]^+^ 666.1906, found 666.1900.

*4β-{4-[1-(β-d-Glucopyranosyloxymethyl)-3,6,9-trioxadec-10-yl]-1,2,3-triazol-1-yl}-4-deoxy-4'-demethylpodophyllotoxin* (**43**). White amorphous powder, yield 92% (after chromatography with CHCl_3_/CH_3_OH, 9:1); mp 104–106 °C; [α]_D_^25.1^: −40.3 (c 0.18, CH_3_OH); ^1^H-NMR (CD_3_OD, 400 MHz): *δ* 7.82 (s, 1H, C^5''^-H), 6.68 (s, 1H, C^5^-H), 6.64 (s, 1H, C^8^-H), 6.38 (s, 2H, C^2'^, C^6'^-H), 6.25 (d, 2H, *J* = 4.8 Hz, C^4^-H), 5.97 (d, 2H, *J* = 5.0 Hz, OCH_2_O), 4.78 (d, 1H, *J* = 4.0 Hz, C^1^-H), 4.62 (s, 2H, C^6''^-CH_2_), 4.31 (d, 1H, *J* = 8.0 Hz, C^1'''^-H), 4.02–3.98 (m, 2H), 3.87–3.79 (m, 2H), 3.74 (s, 6H, C^3''^, C^5''^-OCH_3_), 3.70–3.61 (m, 12H), 3.43–3.39 (m, 2H), 3.28–3.26 (m, 2H), 3.21–3.13 (m, 2H); ^13^C-NMR (CD_3_OD, 100 MHz): *δ* 176.0 (C-12), 150.5 (C-7), 149.2 (C-6) , 148.7 (C-3', C-5'), 145.9 (C-4''), 135.1 (C-9), 131.3 (C-10), 131.3 (C-1'), 126.9 (C-4'), 125.9 (C-5''), 111.2 (C-5), 109.7 (C-8), 109.3 (C-2', C-6'), 104.4 (OCH_2_O), 103.2 (C-1'''), 77.9 (C-5'''), 77.9 (C-3'''), 75.1 (C-2'''), 71.6 (C-4'''), 71.4, 71.4, 70.9 (C-11), 69.6, 68.9, 64.9 (C-6''), 62.7 (C-6'''), 59.8 (C-2), 56.7 (3', 5'-OCH_3_), 44.7 (C-4), 42.7 (C-1), 38.5 (C-3); ESIMS: *m/z* 798 [M+Na]^+^, HRESIMS: calcd for C_36_H_45_N_3_O_13_Na [M+Na]^+^ 798.2692, found 798.2669.

*4β-{4-[1-(β-d-Glucopyranosyloxymethyl)-3,6,9,12,15,18-hexaoxanonadec-19-yl]-1,2,3-triazol-1-yl}-4-deoxy-4'-demethylpodophyllotoxin* (**44**). White amorphous powder, yield 94% (after chromatography with CHCl_3_/CH_3_OH, 9:1); [α]_D_^24.9^: −37.7 (c 0.23, CH_3_OH); ^1^H-NMR (CD_3_OD, 400 MHz): *δ* 7.81 (s, 1H, C^5''^-H), 6.67 (s, 1H, C^5^-H), 6.63 (s, 1H, C^8^-H), 6.38 (s, 2H, C^2'^, C^6'^-H), 6.24 (d, 1H, *J* = 4.7 Hz, C^4^-H), 5.97 (d, 2H, *J* = 5.3 Hz, OCH_2_O), 4.77 (d, 1H, *J* = 4.0 Hz, C^1^-H), 4.64 (s, 2H, C^6''^-CH_2_), 4.35 (d, 1H, *J* = 8.0 Hz, C^1'''^-H), 4.04–4.00 (m, 2H), 3.88–3.78 (m, 2H), 3.75 (s, 6H, C^3''^, C^5''^-OCH_3_), 3.68–3.63 (m, 24H), 3.48–3.39 (m, 2H), 3.31–3.29 (m, 2H), 3.25–3.21 (m, 1H), 3.17–3.11 (m, 1H); ^13^C-NMR (CD_3_OD, 100 MHz,): *δ* 176.0 (C-12), 150.5 (C-7), 149.2 (C-6), 148.7 (C-3', C-5'), 146.0 (C-4''), 136.2 (C-1'), 135.1 (C-9), 131.3 (C-10), 126.9 (C-4'), 125.9 (C-5''), 111.2 (C-5), 109.7 (C-8), 109.3 (C-2', C-6'), 104.4 (OCH_2_O), 103.2 (C-1'''), 77.9 (C-5'''), 77.9 (C-3'''), 75.1 (C-2'''), 71.6 (C-4'''), 71.1, 71.0, 70.9, 70.9 (C-11), 69.6, 68.9, 65.0 (C-6''), 62.7 (C-6'''), 59.8 (C-2), 56.8 (3', 5'-OCH_3_), 44.7 (C-4), 42.7 (C-1), 38.5 (C-3); ESIMS: *m/z* 931 [M+Na]^+^, HRESIMS: calcd for C_42_H_57_N_3_O_19_Na [M+Na]^+^ 930.3478, found 930.3465.

### 3.5. Cell Culture and Cytotoxicity Assay

The following human tumor cell lines were used: HL-60, SMMC-7721, A-549, MCF-7, and SW480. All the cells were cultured in RMPI-1640 or DMEM medium (Hyclone, Logan, UT, USA), supplemented with 10% fetal bovine serum (Hyclone) at 37 °C in a humidified atmosphere with 5% CO_2_. Cell viability was assessed by conducting colorimetric measurements of the amount of insoluble formazan formed in living cells based on the reduction of 3-(4,5-dimethyl- thiazol-2-yl)-2,5-diphenyltetrazolium bromide (MTT) (Sigma, St. Louis, MO, USA). Briefly, adherent cells (100 μL) were seeded into each well of a 96-well cell culture plate and allowed to adhere for 12 h before drug addition, while suspended cells were seeded just before drug addition, both with an initial density of 1 × 10^5^ cells/mL in 100 μL of medium. Each tumor cell line was exposed to the test compound at various concentrations in triplicate for 48 h. After the incubation, MTT (100 μg) was added to each well, and the incubation continued for 4 h at 37 °C. The cells lysed with SDS (200 μL) after removal of 100 μL of medium. The optical density of lysate was measured at 595 nm in a 96-well microtiter plate reader (Bio-Rad 680). The IC_50_ value of each compound was calculated by Reed and Muench’s method.

## 4. Conclusions

In conclusion, we have used an effective and facile Fisher glycosylation strategy to prepare glucose-bearing terminal-alkynes with a catalyst of H_2_SO_4_-silica. Then, all glycosides were subjected to peracetylation or perbutyrylation, and the resulting glycosylated terminal alkynes underwent click-reactions with azide derivatives of podophyllotoxin to yield a series of 4*β*-triazole-linked glucose-podophyllotoxin conjugates in high yields. All conjugated derivatives were screened for anticancer activity against a panel of five human cancer cell lines including HL-60 (leukemia), SMMC-7721 (hepatoma), A-549 (lung cancer), MCF-7 (breast cancer), and SW480 (colon cancer). All these derivatives display different level of anticancer activity which can be affected by the nature of substituents on the glucose residue, the length of the linking spacer between the sugar and the triazole ring, and the substituent on the 4'-position of the E-ring of podophyllotoxin scaffold. Derivatives with a perbutyrylated glucose residue generally display higher anticancer activity than other derivatives. The two most active compounds **29** and **35**, both having a perbutyrylated glucose residue and a 4'-OH on the E ring, are significantly more active than etopodide or cisplatin. Further investigation of these compounds in *in vivo* tumor models is necessary in order to evaluate their therapeutic potential for cancer treatment.
